# Practical Integration of Open-Source Control Architectures on Custom Quadruped Robots: Simulation Validation and Hardware Interfacing

**DOI:** 10.3390/s26154730

**Published:** 2026-07-25

**Authors:** Vishnudev Kurumbaparambil, Subashkumar Rajanayagam, Stefan Twieg

**Affiliations:** Department of Electrical, Mechanical and Industrial Engineering, Hochschule Anhalt, Bernburger Str. 55, 06366 Köthen, Germany

**Keywords:** quadruped robot, open-source control framework, ROS, CHAMP, MIT Mini Cheetah, simulation-to-hardware transition, custom robotics, state machine controller

## Abstract

Custom-built quadrupedal platforms offer an accessible path for robotics research, yet researchers frequently encounter a “framework gap” when porting complex open-source control software to custom hardware. This paper documents the integration and validation process of Scotty, a custom quadrupedal robot, evaluating two distinct open-source control frameworks: the torque-based MIT Mini Cheetah and the position-based CHAMP architectures. Due to minimal documentation, hardware-dependent complexities, and a tightly coupled architecture, it was difficult to achieve a satisfactory result using the MIT framework within our system’s scope. Conversely, CHAMP’s structured integration documentation enabled the deployment of a locomotion pipeline validated in Gazebo simulation alongside a functional hardware interface middle layer. To overcome CHAMP’s lack of native operational state management, we developed a custom state-based controller with a web-based GUI that safely orchestrates transitions across Idle, Ready, Down, Stand, and Walk configurations. While walking was successfully validated in the simulation environment to verify the control software pipeline, physical hardware evaluation was restricted to individual joint control, localized leg movements, and GUI-based parameter tuning. Full closed-loop hardware locomotion was not achieved, as the extensive tuning of gait parameters and controller gains under full system weight was bounded by project constraints. The integration logs, practical hurdles, and architectural lessons documented in this work are shared openly to provide a clear, transferable roadmap for future developers of robotic systems.

## 1. Introduction

Robotics is rapidly advancing into real-world environments, where adaptability, mobility, and robustness are critical. Quadrupedal robots, in particular, offer enhanced stability and terrain coverage, making them ideal for complex and dynamic applications. While commercial robots such as Boston Dynamics’ Spot [1] and Unitree’s Go1 [2] represent the state of the art, their high cost and proprietary hardware and software limit customization and integration. In contrast, custom-built platforms provide full control over both hardware and software, enabling modifications to low-level control, integration of additional sensors, and incorporation of AI modules.

The main challenge in developing custom quadruped robots lies in implementing robust high-level controllers. Open-source frameworks promise to accelerate this process, yet they are typically designed for specific hardware ecosystems. Porting them to custom platforms requires significant reverse-engineering effort, and the “framework gap”, the mismatch between framework assumptions and custom hardware reality, is poorly documented in the literature. This gap forces researchers to discover integration barriers through trial and error, wasting substantial engineering effort.

This paper documents the practical integration process of bringing Scotty, a custom quadruped robot, from assembly to simulation-validated locomotion and joint-level hardware interfacing. We evaluate two fundamentally different open-source frameworks: MIT Mini Cheetah [3,4,5,6,7,8,9,10,11], a low-level torque-based controller, and CHAMP [12,13,14], a high-level ROS-based framework. Our contributions are:

1. A systematic analysis of the low-level architectural assumptions and integration barriers encountered when adapting the tightly coupled MIT Mini Cheetah framework to non-standard hardware ecosystems.
2. The design and implementation of a custom top-level state machine controller featuring an integrated web-based GUI to manage native operational states lacking in the base CHAMP framework.
3. A simulation-based validation demonstrating walking gaits in Gazebo and seamless transitions across operational states to verify the control pipeline.
4. A functional hardware interface layer enabling full-duplex SPI-to-CAN communication, joint-level command mapping, and live parameter telemetry tracking for manual calibration.
5. An open access repository (https://github.com/Vichu95/Scotty) (accessed on 22 July 2026) containing comprehensive hardware integration logs, software configuration files, and troubleshooting documentation to serve as a reproducible roadmap for custom quadruped developers.

## 2. Related Work and Framework Selection

The development of custom mobile robotic platforms spans various locomotion paradigms, each presenting unique structural, kinematic, and control characteristics. These range from wheeled systems optimized for smooth flat terrains to articulated vehicles, passive suspension mechanisms, and legged quadrupeds capable of traversing highly irregular ground. For instance, passive suspensions such as rocker-bogie systems negotiate uneven terrains by passively adapting their link configurations to surface profiles without requiring active torque control at the suspension joints, as analyzed in recent mobility and simulation models [15]. Conversely, legged quadrupedal platforms rely on active closed-loop controllers to coordinate multi-degree-of-freedom (DoF) limb trajectories and manage body pose stability. While active control provides legged systems with superior adaptability, it also introduces a significant computational and hardware-interfacing overhead.

To accelerate development on custom platforms, researchers frequently leverage open-source control architectures. In this study, we evaluate two prominent open-source frameworks: the MIT Mini Cheetah [3] and CHAMP [12]. These frameworks represent two fundamentally different architectural paradigms. The MIT framework utilizes a high-frequency, torque-based control strategy requiring real-time kernels and custom low-level communication interfaces (like SPIne), while CHAMP provides a modular, position-based trajectory planner designed for native ROS-friendly integration. Choosing between these paradigms represents a major design trade-off between peak dynamic performance and software integration simplicity. In Scotty’s development, the MIT framework was initially selected as the academic standard for high-performance quadruped control, while CHAMP was subsequently chosen to provide an accessible, ROS-native integration path when physical hardware constraints bounded the torque-control deployment.

### 2.1. MIT Mini Cheetah Framework

The MIT Mini Cheetah framework [3,4,5,6,7,8,9,10,11] implements a high-frequency, low-level torque control strategy optimized for highly dynamic locomotion. The software ecosystem is designed to run on a Linux kernel enhanced with a PREEMPT-RT patch [16,17] to guarantee deterministic execution. The architecture is organized into a modular common library, a rigid-body dynamics simulator, a robot abstraction framework, and user controller code. Inter-process communication is handled via Lightweight Communications and Marshalling (LCM) at a frequency of 1 kHz [18]. The system architecture uses an intermediate communication interface called SPIne [5], featuring two STM32F446 microcontrollers on the SPI bus, each generating two CAN networks for actuator communication.

However, the framework suffers from minimal documentation, tightly coupled software components, and assumptions about specific hardware ecosystems. The MIT Mini Cheetah actuators have internal microcontrollers running PD position and velocity control loops at 40 kHz. The dynamics are modeled using rigid body principles, with actuator dynamics playing a critical role. While powerful, this framework is designed for a specific ecosystem, making adaptation to custom hardware particularly challenging.

### 2.2. CHAMP Framework

CHAMP [12,13] is an open-source ROS-based development framework for quadrupedal robots. Unlike MIT Mini Cheetah, CHAMP provides documentation, a structured approach, and a setup assistant [14] for configuring custom robots. It offers native ROS compatibility, modular architecture, Gazebo support for simulation, and pre-configured URDFs for well-known quadrupedal robots including ANYmal, MIT Mini Cheetah, and Boston Dynamics Spot, among others. The framework generates both simulation and hardware-related files automatically after robot configuration.

The core computational input for CHAMP is the robot’s Unified Robot Description Format (URDF) model. By leveraging the URDF data, CHAMP automatically isolates link transformations, maps inverse kinematics matrices, and configures pre-mapped simulation environments in Gazebo and RViz. Furthermore, if the base URDF encompasses sensor links (such as LiDAR or depth cameras), the framework can autonomously generate layout launch files for Simultaneous Localization and Mapping (SLAM). To port CHAMP commands to actual physical hardware, developers must construct a dedicated hardware interface layer that translates published ROS joint trajectory topics into motor commands while streaming physical actuator feedback states back to the high-level ROS control loop.

Recent open-source efforts have further expanded the ROS ecosystem for legged platforms. For example, Hyperdog [19] is an open-source quadruped robot platform based on ROS2 and micro-ROS, demonstrating how modern middleware and structured software layers can simplify low-level motor interfacing and coordinate transforms on custom hardware. In this context, CHAMP’s structured integration assistant offers a mature, ROS1-native pipeline that generates simulation and hardware configurations directly from a robot’s Unified Robot Description Format (URDF) description, allowing developers to isolate joint-level control and test software pipelines.

### 2.3. Framework Selection Rationale

Our initial selection of MIT Mini Cheetah was motivated by its dynamic performance and direct torque control paradigms. However, hardware deployment attempts revealed acute implementation barriers within a custom robot setup. These included undocumented coordinate transformation frames, structural simulation-to-hardware mismatches, and severe configuration complexities within the low-level embedded communication layers that hindered reliable full-system synchronization within the project’s initial scope. Resolving these embedded constraints within a restricted development scope proved highly inefficient.

CHAMP was selected as our primary operational framework because its native ROS compatibility, extensive documentation, and decoupled modularity drastically reduced integration complexity on custom hardware. While position-based trajectory control offers less explosive agility compared to pure torque-driven approaches, its structural flexibility enabled successful validation of the software stack and provided an accessible foundation for parameter tuning. A comparative summary of both architectures, focusing on qualitative, experience-based integration metrics derived from our development trials, is outlined in Table 1.

## 3. Robot Platform: Scotty

### 3.1. Mechanical Design and Physical Metrics

**Figure 1:**
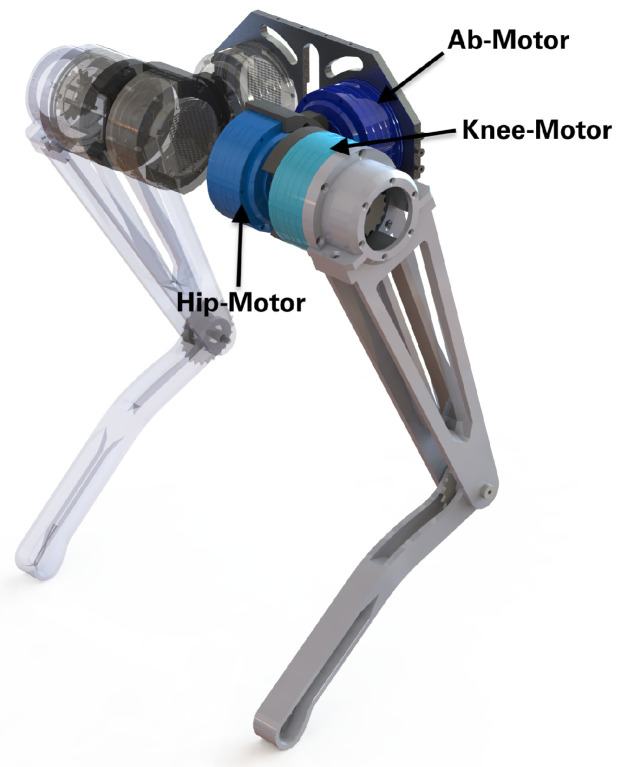
CAD rendering of Scotty’s single leg showing the three active degrees of freedom: abduction/adduction (abad) motor, hip motor, and knee motor with integrated chain-and-gear transmission. All joints are actuated by CubeMars AK10-9 brushless DC motors.

The mechanical design of Scotty focuses on structural modularity and robust physical balance, enabling streamlined maintenance and incremental component testing. The platform features four symmetrically arranged legs, each possessing three active degrees of freedom (DoF) to replicate natural quadrupedal gait kinematics. The primary structural links (Figure 1) composing each limb are:

1. The **abduction/adduction (abad) joint**, located at the base of the lateral shoulder frame, which regulates side-to-side hip motion and weight-shifting balance.
2. The **hip joint**, positioned on the upper leg assembly, which governs the forward and backward rotational sweep of the thigh to drive horizontal propulsion.
3. The **knee joint**, which regulates lower leg bending and ground-contact extension via an integrated chain and gear transmission mechanism featuring a 24:16 teeth sprocket ratio (yielding a 1.5× gear reduction scaling).

**Figure 2:**
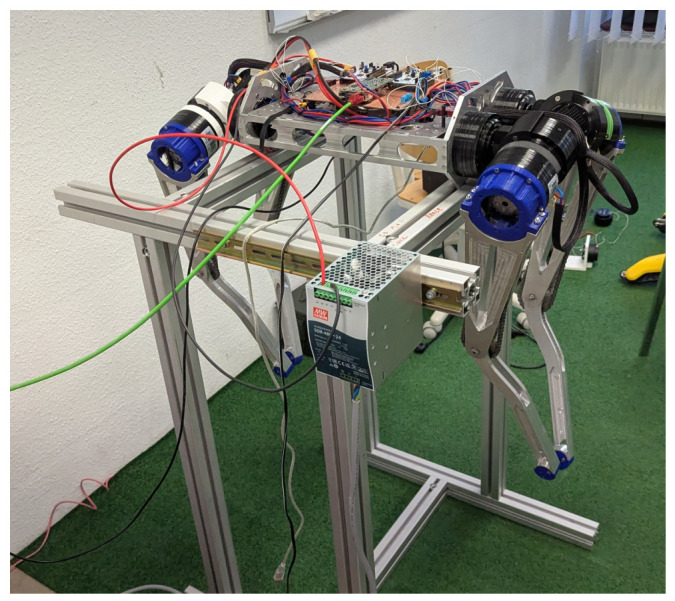
Photograph of the fully assembled Scotty quadruped robot showing the aluminum chassis, four symmetric legs with 3-DoF each, and internal wiring routed through aluminum backplates. The platform measures 400 mm × 250 mm × 150 mm and weighs 19.31 kg.

The robot’s chassis and limb segments are primarily machined from lightweight aluminum to withstand significant operational torques. The main body envelope measures approximately 400 mm in length, 250 mm in width, and 150 mm in height. While the total mass properties calculated from the baseline Computer-Aided Design (CAD) model initially estimated 20.25 kg, stripping away unmounted peripheral components (such as specialized depth cameras and LiDAR arrays) for this experimental stage established an exact physical platform weight of 19.31 kg. Internal routing paths within the aluminum backplates shield the wiring networks from structural interference during maximum leg flexion and extension (Figure 2).

A comprehensive overview of Scotty’s physical, kinematic, and electrical parameters is compiled in Table 2, based on SolidWorks 2022 CAD mass calculations and manufacturer datasheets.

To verify the feasibility of static standing and ensure the actuators operate within safe thermal limits, a simplified torque margin assessment was conducted. Given the robot’s physical mass of 19.31 kg, symmetric static standing distributes the weight equally among all four limbs, requiring each leg to support approximately 47.4 N of vertical force. At a nominal standing height of 0.48 m, the hip and knee joint torques required to maintain this posture are estimated to be well below the CubeMars AK10-9 actuator’s continuous rated torque limit of 18.0 Nm (and peak torque limit of 48.0 Nm). This provides a continuous safety margin of more than 2.5× under static conditions. It is important to emphasize that this torque margin assessment is a simplified static estimate rather than a proof of dynamic locomotion feasibility, as dynamic maneuvers introduce significant inertial forces, foot impact loads, and foot–ground interaction dynamics that can dramatically increase joint torque requirements beyond these static baselines. Nonetheless, this static calculation confirms that the physical actuators are more than sufficient to support the robot’s self-weight during standing and support phases without risking thermal overload.

Regarding the kinematic parameters, the physical leg links have a total combined length of 0.655 m when fully extended (0.3175 m thigh and 0.3375 m shin). In the CHAMP software configuration, the default walking height parameter (clearance) was set to 0.50 m, and the nominal standing height was set to 0.48 m. This operating height range (0.48–0.50 m) represents approximately 73% to 76% of the maximum leg reach, which is a deliberate kinematic design choice. It maintains a moderately bent leg posture during standing and walking, avoiding kinematic singularities (locked straight joint limits at 0.655 m) while providing sufficient joint torque leverage and vertical swing clearance. Although a standing height of 0.50 m is relatively tall compared to the chassis length (0.40 m), this aspect ratio is physically dictated by the long link dimensions required to clear obstacles in experimental environments.

### 3.2. Sensors and Actuators

Scotty’s locomotion is driven by twelve AK10-9 V2.0 KV60 brushless DC motors sourced from CubeMars [20]. These actuators operate across a 24–48 V power envelope and are capable of outputting a peak maximum torque of 48 Nm. The internal motor controllers are based on the MIT Mini Cheetah hardware design [5,11], offering advanced control capabilities and supporting two primary communication protocols. During continuous robot operation, the actuators communicate exclusively via a high-speed CAN bus network. Initial configuration, joint calibration, and parameter tuning are performed over a UART connection using the manufacturer’s R-Link V2.0 hardware interface. This standalone interface allows developers to map unique CAN IDs, establish low-level safe current limits, fine-tune control gains, and execute precise motor position zeroing procedures prior to hardware deployment.

Real-time state estimation and orientation tracking are achieved using a VectorNav VN-100 industrial-grade Inertial Measurement Unit (IMU) [21]. This sensor combines a 3-axis accelerometer, a 3-axis gyroscope, and a 3-axis magnetometer alongside an internal 32-bit processor running proprietary calibration algorithms to filter sensor drift and local electromagnetic interference. The IMU is rigidly mounted to the center of the main printed circuit board layer to ensure accurate tracking of the chassis pitch, roll, and raw directional acceleration vectors. In this stage of Scotty’s physical integration, the IMU was utilized exclusively for orientation logging and raw directional acceleration telemetry streaming over ROS. It was not integrated into the active closed-loop feedback path for walking stabilization, since locomotion on the physical hardware was restricted to joint-level open-loop execution. Sensor fusion algorithms (such as Kalman filtering on the IMU outputs) were validated in Gazebo simulation but were bypassed in physical testing to isolate low-level communication and command mapping issues.

### 3.3. Computing and Communication Architecture

The computing stack uses a decentralized hierarchical control topology to balance intensive kinematic tracking against deterministic low-level communication cycles. High-level trajectory generation and operational state tracking are executed on an x86-based UP Board (UP-CHT01) [22] single-board computer running a real-time patched Linux kernel (PREEMPT-RT) [16,17]. Low-level motor command delivery and feedback data parsing are distributed across two independent STM32F446RE ARM Cortex-M4 microcontrollers [23], which serve as intermediate limb controllers.

**Figure 3:**
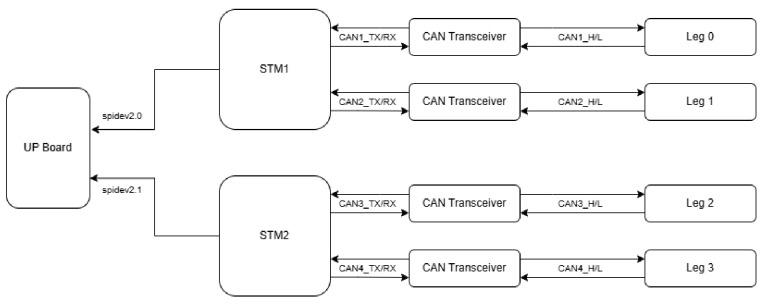
Hierarchical communication topology showing the UP Board as SPI master communicating with two STM32F446RE microcontrollers via dedicated SPI buses. Each STM32 slave controls two legs via independent CAN transceivers, with each leg operating on a dedicated CAN bus supporting three actuators (abduction, hip, knee). This topology creates four parallel CAN networks for deterministic low-level motor control.

The internal messaging network layout (Figure 3) is structured as follows:

- **Master-to-Slave SPI Network:** The UP Board operates as a full-duplex SPI master interface, establishing concurrent communication pipelines with the two STM32 slaves mapped to dedicated system nodes (/dev/spidev2.0 and /dev/spidev2.1).
- **Slave-to-Actuator CAN Network:** Each low-level STM32 microcontroller governs two independent legs using dual hardware CAN transceivers. To optimize bus bandwidth and eliminate latency bottlenecks, each leg operates on an isolated, dedicated CAN bus path supporting three daisy-chained joint actuators (Abad, Hip, and Knee), resulting in four parallel CAN networks across the entire system topology.

**Figure 4:**
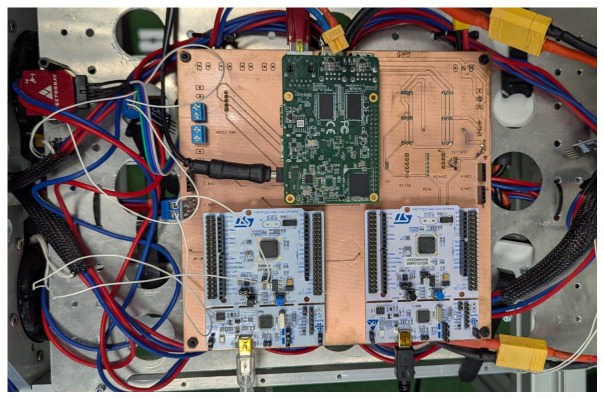
Custom two-layer printed circuit board.

A custom two-layer Printed Circuit Board (PCB) (Figure 4) serves as the primary backplane, power distribution grid, and routing medium, significantly minimizing cable clutter, stabilizing voltage regulation, and allowing the low-level microcontrollers to be safely extracted during modular hardware diagnostics.

**Figure 5:**
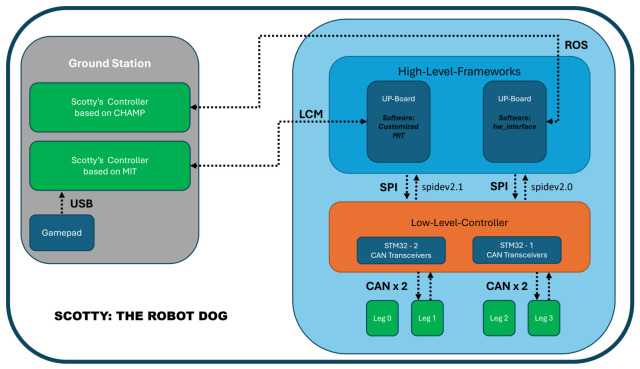
Complete system architecture showing the dual-framework ground station (CHAMP-based and MIT-based controllers), high-level ROS frameworks on the UP Board, and low-level STM32 controllers. The architecture spans three layers: (1) ground station with gamepad input and USB interface; (2) high-level frameworks with software customization layer and web interface; (3) low-level controllers with STM32 microcontrollers, CAN transceivers, and four leg groups connected via SPI and CAN buses.

A simplified architecture overview is shown in Figure 5.

## 4. MIT Mini Cheetah Framework: Integration Attempt and Lessons

### 4.1. Integration Approach and Low-Level Optimizations

**Figure 6:**
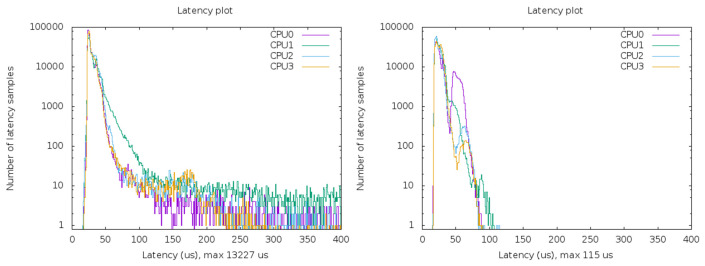
Latency histograms comparing standard Linux kernel (**left**, max latency 13,227 μs) versus PREEMPT-RT patched kernel (**right**, max latency 115 μs) on the UP Board.

The primary phase of the software implementation focused on porting the core MIT Mini Cheetah control framework onto Scotty’s high-level UP Board computer. Initial platform adjustments included installing a pre-compiled Linux kernel with a PREEMPT-RT patch [16,17], reducing worst-case processing latencies from 13,227  μs to a deterministic 115 μs (Figure 6). System peripheral nodes were explicitly mapped within the high-level codebase, routing the IMU to /dev/ttyS4 and setting up static IP network protocols to enable stable, low-latency remote execution via Secure Shell (SSH) connections.

To bridge the high-level control code with the low-level limb actuators, several structural modifications were integrated directly into the intermediate STM32 microcontroller firmware layer:

- **Firmware SPI Error Callbacks:** Within the low-level STM firmware, dedicated re-initialization routines were embedded directly into the SPI peripheral error callbacks (HAL_SPI_ErrorCallback). This design forcibly reinitializes the communication module in interrupt mode following data link drops, bypassing manual hardware resets and ensuring continuous streaming connectivity.
- **CAN Bus Inter-Message Timing:** In accordance with architectural recommendations from Ben Katz’s research, a precise 300 μs execution delay was introduced between consecutive outgoing CAN messages. This timing adjustment accommodates physical bus propagation and prevents transmission packet collisions across the multi-actuator network layout.
- **Dual Range Separation Limits:** To prevent severe data misinterpretations during float-to-integer conversion packing, the STM firmware decouples localized scaling constraints from global actuator boundaries. Local safety thresholds (e.g., limiting torque dynamically to a restricted range of ±2 Nm) are applied within a localized software layer, while the mapping equations retain the global native motor scaling ranges (±65 Nm) to accurately output correct raw 12-bit unsigned integer commands via the CAN bus line.
- **Motor Control Mode Sequence Management:** The firmware explicitly handles sequential mode transitions by feeding specific CAN code frames directly to the internal actuator drivers. A systematic command sequence analysis established that initializing the motors requires issuing an ENTER motor control command, followed by a localized ZERO command sequence to calibrate the vertical physical position reference baseline, and an EXIT frame to cleanly terminate the high-torque mode loops.

### 4.2. Encountered Integration Challenges

#### 4.2.1. Simulation-to-Hardware Spatial Mismatch

**Figure 7:**
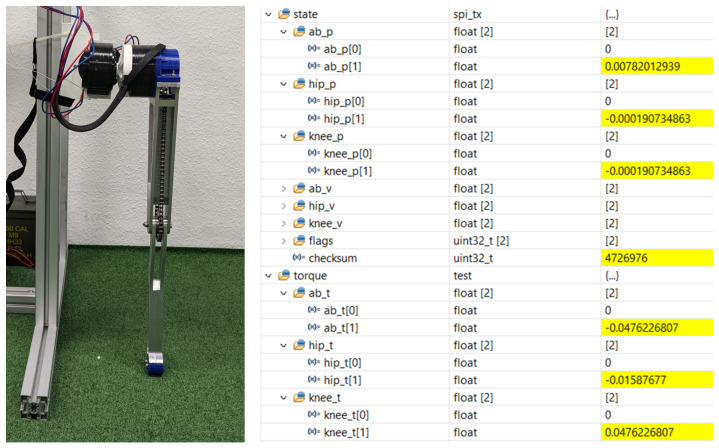
(**Left**) Physical leg assembly aligned vertically downward for zero-position calibration, matching the MIT framework’s kinematic assumption that 0 rad corresponds to limbs oriented perfectly vertical. (**Right**) Live telemetry display showing calibrated joint positions (abduction, hip, knee) and torque values after zeroing procedure.

During initial full-system tests, acute synchronization mismatches were observed between the simulated visual environment and the physical leg configurations. Actuator movements frequently executed in inverted directions relative to the software model. Systematic tracing of the high-level communication architecture revealed that the underlying control laws operate under a rigid spatial assumption: the zero-position configuration (0 rad) corresponds to the limbs oriented perfectly vertical downward (Figure 7). Consequently, manual hardware alignment sequences and calibration offsets had to be hardcoded within the translation files to unify the physical reference frames with the software kinematics.

#### 4.2.2. Motor Direction Mapping

Due to the complete absence of official MIT documentation outlining expected robot orientations or default motor rotation directions, the open-source MiLAB adaptation [24] provided the only viable baseline architectural reference. To verify this baseline against Scotty’s physical hardware, each motor’s positive rotation axis was empirically determined through isolated, single-joint physical actuation testing.

**Figure 8:**
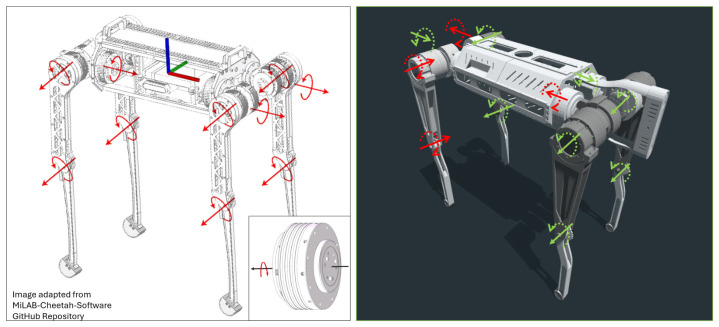
(**Left**) MIT Mini Cheetah coordinate frame according to the MiLAB repository, showing expected positive rotation axes for all twelve joints. (**Right**) Scotty’s actual motor mounting orientations revealing four joints with inverse rotational directions compared to the MIT baseline. Red arrows indicate axes where physical rotation direction is opposite to the framework’s assumed positive direction.

Comparing these experimental motor telemetry vectors against the framework’s assumed orientations revealed that four of Scotty’s joint installations generated inverse rotational metrics (Figure 8). Direction compensation for these inverted axes was initially executed as a firmware patch within the low-level microcontroller code. However, subsequent structural refinements required migrating this correction layer to handle axis inversions exclusively at the high-level layer. This migration was essential to honor MIT’s core architectural intent, which mandates that coordinate frames and kinematic transformations be managed centrally rather than distributed across embedded sub-systems.

#### 4.2.3. Transmission Gear Ratio Scaling

Unlike the direct-drive design paradigms common in specific research quadrupeds, Scotty utilizes a secondary sprocket and chain assembly to actuate the knee joints. Featuring a 24:16 teeth profile, this configuration introduces a distinct mechanical reduction ratio:

$$\mathrm{Gear}\;\mathrm{Ratio}=\frac{24}{16}=1.5$$

Because the high-level framework expects feedback and control parameters relative to the final knee link position rather than the raw motor output shaft, bidirectional scaling loops were introduced into the software data pipeline:

- **Position and Velocity Scaling:** Outgoing high-level position and velocity commands are multiplied by 1.5 before transmission to the low-level controllers ($q_{\mathrm{motor}\_\mathrm{cmd}}=1.5\cdot q_{\mathrm{joint}\_\mathrm{des}}$ and ${\dot{q}}_{\mathrm{motor}\_\mathrm{cmd}}=1.5\cdot {\dot{q}}_{\mathrm{joint}\_\mathrm{des}}$). Conversely, incoming motor encoder feedback is divided by 1.5 to yield the actual joint-level position and velocity state ($q_{\mathrm{joint}}=q_{\mathrm{motor}}/1.5$ and ${\dot{q}}_{\mathrm{joint}}={\dot{q}}_{\mathrm{motor}}/1.5$).
- **Torque and Feed-forward Scaling:** Due to mechanical advantage, the torque at the knee joint is amplified by the reduction ratio. Therefore, outgoing feed-forward joint torque commands must be divided by 1.5 before being sent to the motor driver ($\tau_{\mathrm{motor}\_\mathrm{cmd}}=\tau_{\mathrm{joint}\_\mathrm{des}}/1.5$). Correspondingly, raw motor telemetry torque feedback is multiplied by 1.5 to calculate the actual torque acting on the leg link ($\tau_{\mathrm{joint}}=1.5\cdot \tau_{\mathrm{motor}}$).

#### 4.2.4. Rigid-Body Dynamics Configurations

To align the framework’s predictive rigid-body models with Scotty’s physical reality, the baseline parameters within the MIT dynamics header file (MiniCheetah.h) were systematically updated with structural properties extracted directly from the SolidWorks CAD assembly mass property tools. The global chassis mass parameter was set to 19.31 kg, reflecting the physical prototype stripped of unmounted peripheral sensors, with the structural body envelope configured to dimensions of 400 mm (length) × 250 mm (width) × 150 mm (height). The 3×3 spatial rotational inertia tensors (in units of kg·$m^{2}$) and centers of mass (COM, in meters) were calculated for each independent link segment. The coordinate frame origins for these parameters were defined in alignment with the respective parent joint rotation axes, matching standard URDF coordinate frame conventions.

Actuator properties were updated to mirror the specifications of the CubeMars AK10-9 units, defining an internal planetary gear reduction ratio of 9:1, a nominal bus voltage baseline of 24 V, and a torque constant ($K_{t}$) of 0.198 Nm/A [20]. The rotor mass was modeled at 0.192 kg (constituting approximately 20% of the total motor mass), and its rotational inertia around the spinning axis was mapped to the native tensor fields using the manufacturer-specified value of $1.002\times 10^{-4}$ kg·$m^{2}$ (1002 g·${\mathrm{cm}}^{2}$). Finally, to capture non-linear joint losses and ensure numerical solver stability, joint damping and dry friction coefficients were introduced. Because experimental dyno measurements of link-level friction were not conducted, these parameters were selected empirically: the joint damping coefficient was set to 0.01 N·m·s/rad, and the joint dry friction baseline was defined as 0.20 N·m within the software configuration.

#### 4.2.5. SPI Bus Line Transmission Collisions

**Figure 9:**
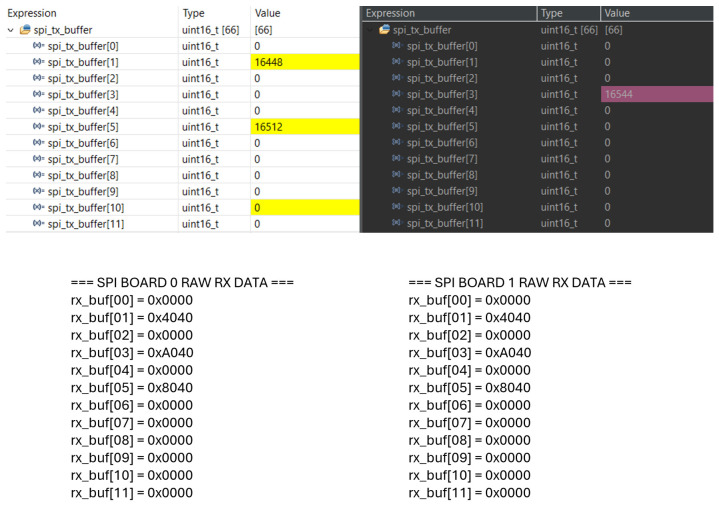
(**Top**) SPI transmit buffer values showing intended data. (**Bottom**) Raw RX data buffers from both SPI boards demonstrating bitwise OR corruption: when one STM32 transmits 0x4040 and another concurrently sends 0x40A0, the master reads 0xA040 due to both slaves simultaneously driving the shared MISO line. This was resolved by reconfiguring SPI to hardware slave-select mode with automatic MISO tri-stating.

When executing control states simultaneously across all limbs, persistent checksum failures occurred within the communication interface, generating bad checksum logs on the high-level processor. Diagnostic telemetry capture from the data processing buffers revealed that both low-level STM32 microcontrollers were driving the shared SPI Master In Slave Out (MISO) line concurrently. This concurrent bus access resulted in an unintended bitwise OR signal corruption across the shared bus channel. For instance, as verified via live telemetry capture, when one microcontroller transmitted 0x4040 and the other concurrently pushed 0x40A0, the master read a corrupted, byte-swapped value of 0xA040, breaking the data package integrity layout (Figure 9).

To resolve this conflict, the low-level communication pipeline was completely re-architected within the STM firmware to decouple transmission timing. The main background thread was restricted to managing time-sensitive CAN bus transmission loops, while the SPI data framework was migrated to run entirely on hardware interrupts via the non-blocking HAL_SPI_TxRxCpltCallback routine.

Crucially, the hardware peripheral setup was updated inside the initialization function (MX_SPI1_Init) to transition from soft slave management to strict hardware protocols by mapping the structural parameter:

hspi1.Init.NSS=SPI_NSS_HARD_INPUT

The physical microcontroller pin PA15 was remapped to alternate function mode inside the general purpose configuration block (MX_GPIO_Init). This configuration forces the internal hardware registers to automatically tri-state the MISO line into a high-impedance state whenever a specific controller’s chip-select line goes inactive, completely isolating the bus lines and enabling successful independent data streaming across all legs.

#### 4.2.6. Oscillatory Instability

**Figure 10:**
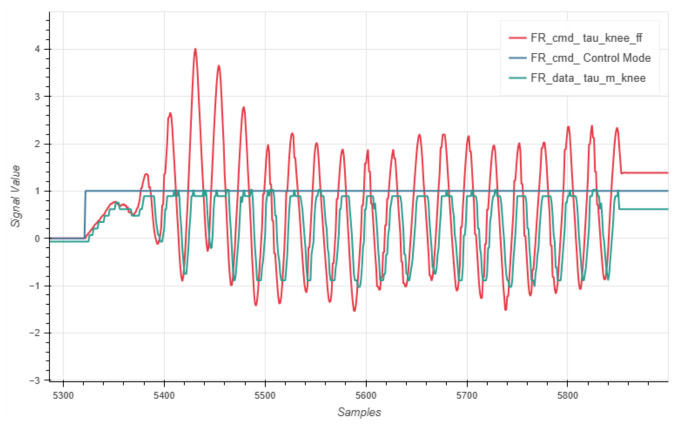
Torque tracking plot showing severe vibrational resonance during MIT framework standing control on the physical robot. The commanded knee torque (red, FR_cmd_tau_knee_ff) and the actual motor torque (green, FR_data_tau_m_knee) exhibit high-frequency oscillations, and the control mode (blue, FR_cmd_ControlMode) shows rapid Mode switching. The instability occurs because the state estimator assumes a stationary ground contact while the chassis is suspended in a safety frame, causing the Kalman filter to diverge and trigger continuous controller overcorrections.

When transitioning from initialization to active standing control, the physical limbs exhibited severe structural vibrations accompanied by rapid torque output fluctuations, as seen in Figure 10. While structural flexibility of the safety test rig, joint backlash, and minor actuator command latencies may have contributed to this resonance, the primary driver is highly likely to have been the mismatch between the suspended physical testing conditions and the closed-loop state estimation and control assumptions embedded in the MIT framework:

- **Ground Force Estimation vs. Measurement:** The MIT framework does not measure ground reaction forces directly (as Scotty lacks tactile foot sensors); instead, contact state is estimated heuristically based on gait scheduling and kinematic residuals. The convex MPC controller solves for optimal ground reaction forces $f_{\mathrm{grf}}$ to support the torso, mapping them to target joint torques via the Jacobian transpose: $\tau =J^{T}f_{\mathrm{grf}}$.
- **Kalman Filter State Estimator Divergence:** The core state estimator uses a Kalman filter to track the robot’s base position and velocity by fusing IMU orientation with leg kinematics. When a foot is scheduled to be in contact with the ground (stance phase), the estimator enforces the constraint that the foot velocity relative to the ground is zero (${\dot{p}}_{\mathrm{foot}}=0$). However, because Scotty was suspended in a safety rig, the feet moved unhindered in mid-air. When the controller commanded joint torques τ, the limbs accelerated rapidly in the absence of opposing ground forces. This kinematic violation caused the Kalman filter velocity estimates to diverge rapidly, reporting false, high-frequency base attitude and velocity drifts.
- **Whole-Body Control Overcorrection:** The high-level Whole-Body Control (WBC) and MPC loops perceived these estimator drifts as massive external disturbances. To compensate, the loops commanded rapid, maximum-effort torque corrections, causing the limbs to enter a violent, self-exciting oscillatory cycle.
- **Mismatched Gain Calibration:** The default framework gains were calibrated for the original MIT Mini Cheetah, which features extremely light, direct-drive shin links (approx. 0.15 kg). In contrast, Scotty’s hardware utilized a significantly heavier knee link (approx. 0.52 kg) and a 1.5× chain-and-sprocket reduction mechanism. When the physical test was executed under Mode 1 with proportional gain $K_{p}=25.0$ N·m/rad and derivative gain $K_{d}=1.0$ N·m·s/rad, the high stiffness gains coupled with the larger inertia and chain backlash directly excited mechanical resonance.

### 4.3. Testing Results and Diagnostic Analysis

System validation was executed incrementally using a custom logging setup. Because the native framework lacked an integrated method for streaming physical torque vectors, a specialized data aggregation pipeline was constructed within the high-level processor to parse the telemetry stream. The 32-bit integer communication flag field was segmented, allowing the three motor torque values (initially compressed into 10-bit floats spanning ±70 Nm) and the active control mode states to be embedded and passed seamlessly through the runtime stack for comprehensive CSV logging and visualization.

**Figure 11:**
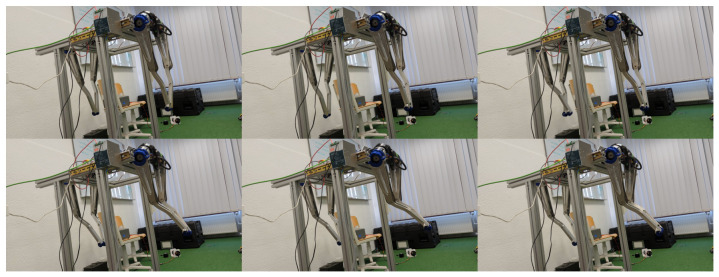
Time-lapse sequence showing Scotty during Mode 0 (Initialization) with the MIT framework.

The empirical behaviors across the primary operational modes are detailed below:

- **Mode 0 (Initialization):** Initial testing revealed that default framework parameters induced wide link extensions, threatening structural collisions with the laboratory testing frame. To guarantee a safe initialization sequence, the configuration file (initial_jpos_ctrl.yaml) was modified to command a uniform $40^{\circ }$ forward knee bend target vector. Under this layout, the joints successfully converged to their designated starting coordinates with active Proportional–Derivative ($K_{p}$, $K_{d}$) gains. However, tests initiated with backwards knee-flexion configurations encountered data clamping bottlenecks; the low-level STM firmware strictly enforced asymmetrical soft joint constraints, restricting the final command outputs and leading to unresponsive joints. This was stabilized by implementing extended, symmetrical testing limits across the code base. Figure 11 shows the snapshots of the robot during Mode 0.
- **Mode 1 (Standing):** Upon engaging the standing loop, the limbs developed rigid resistance, validating the delivery of targeted joint torques. However, the physical limbs immediately entered a state of severe vibrational resonance rather than holding a constant, static torque configuration. Increasing the torque threshold limits within the microcontroller safety functions to mitigate potential saturation did not eliminate the issue, which instead became marginally more acute. This confirmed that the instability was rooted in ungrounded chassis dynamics, as the aggressive default $K_{p}$ and $K_{d}$ gains amplified corrections in the absence of opposing ground reaction forces.

Due to the structural oscillations and overcorrections logged during suspended validation stages, the platform was never deployed on the ground for full body-weight support. Ground-contact testing was deliberately withheld as a critical safety precaution. Because the brushless DC actuators (CubeMars AK10-9) are highly powerful and capable of generating dynamic torque spikes up to 48 Nm, initiating ground-contact closed-loop trials without full confidence in the stability of the state estimator and controller gains presented a significant safety hazard to the operators and a risk of catastrophic structural damage to the custom prototype.

### 4.4. Lessons for the Community

For researchers considering the adaptation of the MIT Mini Cheetah framework to custom hardware platforms, a significant reverse-engineering overhead must be anticipated. The architecture is intrinsically optimized for a highly specific proprietary ecosystem. Achieving operational success requires either replicating that ecosystem identically or executing extensive low-level firmware re-architecting. The key engineering lessons derived from our integration attempt include:

1. **Empirical Zero-Position and Gear Scaling Calibration:** Actuator zero positions cannot be assumed out of the box and must be physically locked with the limbs oriented perfectly vertical downward before issuing alignment frames. Furthermore, non-direct drive systems, such as Scotty’s 1.5× knee sprocket-and-chain transmission, mandate that bidirectional coordinate scaling arithmetic be strictly enforced within the data pipeline to prevent spatial tracking loops.
2. **Empirical Verification of Directional Axes:** Coordinate frame conventions must be derived through systematic single-joint physical testing rather than relying solely on simulation models or secondary documentation. Moreover, direction compensation should be integrated centrally within the high-level kinematic abstraction layers rather than distributed across embedded sub-systems to maintain architectural compliance.
3. **Bridge Software Architecture Gaps with High-Precision Logs:** Transitioning from simulation models to actual physical hardware requires intermediate validation stages utilizing high-precision telemetry. Standard float-to-CSV logging configurations (e.g., %f) induce rounding errors that alter the IEEE 754 binary representation of state vectors, provoking persistent checksum failures upon data unpack re-entry. Developers must enforce high-precision parsing formats (e.g., %.9g) to preserve binary integrity across processing layers.
4. **NaN Detection and Exception Handling:** Faulty dynamics computations or simulator unresponsiveness in the high-level controller can propagate NaN values through the command pipeline. The low-level firmware must implement explicit validation checks that scan incoming SPI data structures for non-numeric values before committing updates to motor command registers. In our implementation, a dedicated check_nan_in_spi_rx() routine iterates across all floating-point fields in the received command packet; if any NaN is detected, the entire frame is rejected and the motors retain their last valid state. This safeguard prevented multiple potential runaway torque scenarios during MIT framework testing.
5. **Multi-Tier Clamping and Range Safeguards:** Joint limits and torque overrides must be implemented independently across multiple hierarchical software layers (actuator internal drive, low-level microcontroller, and high-level control code), ensuring that a single software fault cannot bypass all safety boundaries.
6. **Decoupled Processing Hooks for Incremental Debugging:** The tightly coupled nature of the native framework complicates localized troubleshooting. Firmware design should explicitly isolate background CAN communication loops from interrupt-driven SPI buses. Additionally, introducing dedicated live expression tracking variables (e.g., an exit_command loop hook) ensures that active testing cycles can be terminated gracefully, sending safe exit codes to the actuators and cleanly closing communication lines.
7. **Incremental Gain Tuning from Conservative Baselines:** When adapting torque-based controllers to custom hardware with different mass distributions and actuator responses, default framework gains are rarely transferable. We recommend initiating parameter tuning from deliberately conservative (low) $K_{p}$ and $K_{d}$ values, then incrementally increasing stiffness and damping while monitoring for vibrational resonance. This approach should first be validated under suspended or reduced-load conditions before transitioning to full ground-contact testing. In our MIT integration attempt, the default configuration’s aggressive gains-calibrated for the original Mini Cheetah’s lighter, direct-drive limbs-amplified oscillatory behavior when applied to Scotty’s heavier, chain-driven knee mechanism. A structured, incremental tuning protocol would have isolated this mismatch earlier and reduced hardware risk.

We document our complete engineering log, standalone C++ validation framework, and localized troubleshooting metrics within our open access repository (https://github.com/Vichu95/Scotty, accessed on 22 July 2026) to accelerate future community integration efforts.

## 5. CHAMP Framework: Architecture and Implementation

### 5.1. URDF Generation and Configuration Setup

#### 5.1.1. SolidWorks to URDF Export

**Figure 12:**
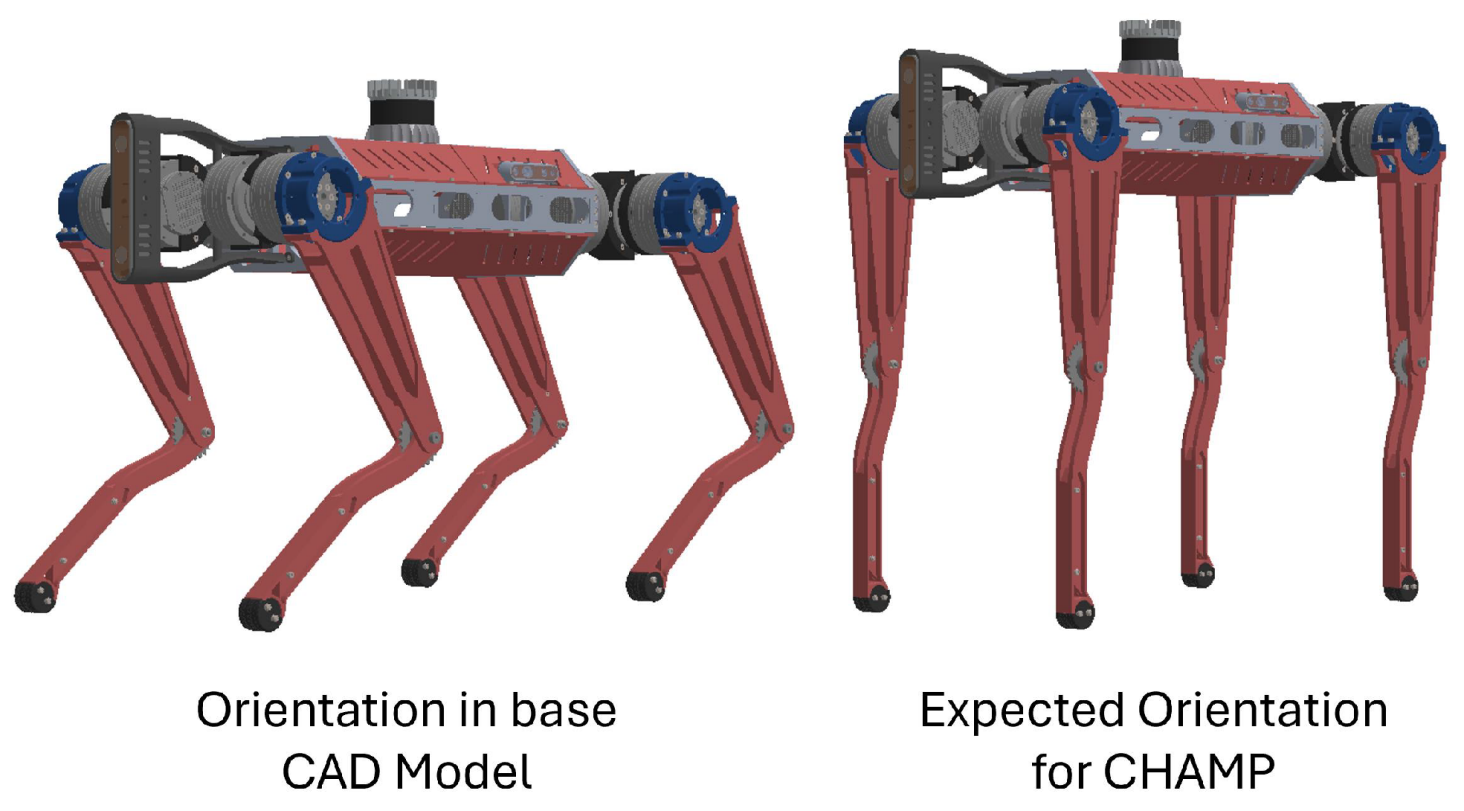
Comparison of Scotty’s base CAD model orientation (**left**, legs in natural resting pose) versus the required zero-position configuration for CHAMP framework integration (**right**, all legs perfectly vertical downward). The exported URDF requires explicit geometric alignment to CHAMP’s expected reference baseline before export to ensure correct inverse kinematics and gait generation.

**Figure 13:**
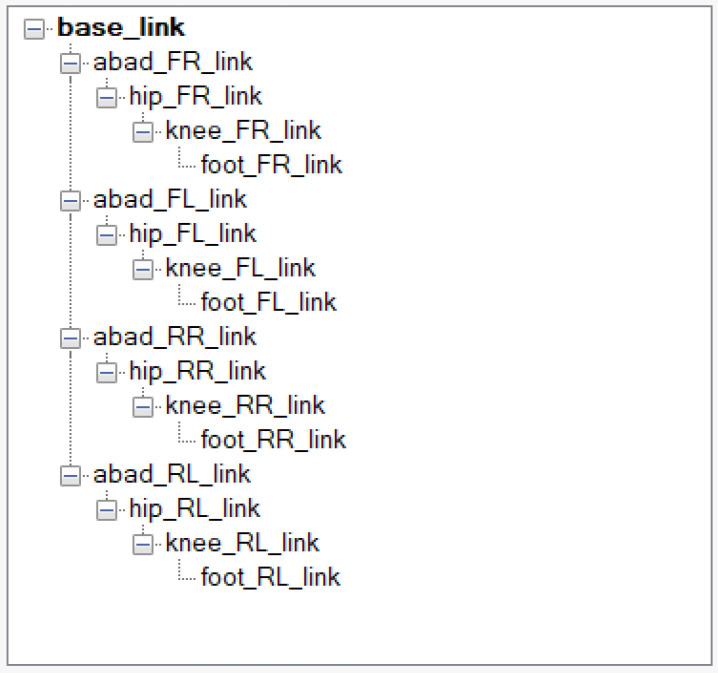
Kinematic tree structure extracted from the SolidWorks URDF export plugin showing the parent–child link hierarchy for all four legs.

**Figure 14:**
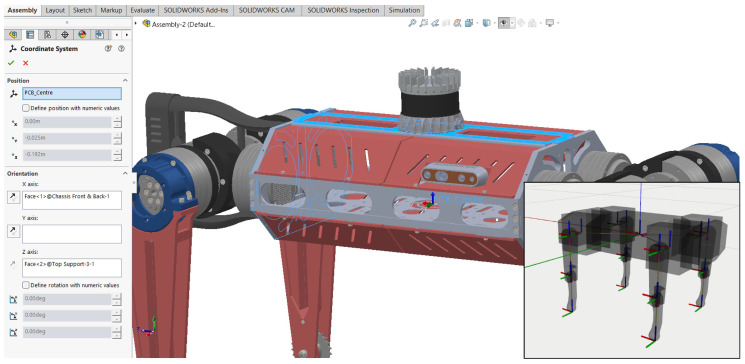
SolidWorks coordinate system configuration interface showing the global reference frame assignment for the URDF export. The main torso (base_link) serves as the global center node, with localized coordinate origins and rotation axes assigned to each individual joint. The inset shows the expected coordinate frame visualization from CHAMP.

The implementation of the CHAMP framework initiated with translating Scotty’s physical dimensions and inertial properties from the Computer-Aided Design (CAD) space into a structured Unified Robot Description Format (URDF) file compatible with the Robot Operating System (ROS) ecosystem. This migration process utilized the SolidWorks URDF Exporter plugin [25]. To guarantee proper coordinate tracking, the export sequence was broken down into a systematic four-part process:

1. **Geometric Alignment:** Positioning all legs perfectly vertical downward to match CHAMP’s expected zero-position configuration reference baseline, as illustrated in Figure 12;
2. **Kinematic Tree Definition:** Structuring the precise parent–child link hierarchy mapped across every limb: base_link→abad→hip→knee→foot, ensuring a seamless kinematic chain propagation (Figure 13);
3. **Coordinate Systems Designation:** Assigning localized coordinate origins and reference rotation axes for each individual joint, keeping the main torso as the global center node (Figure 14);
4. **Joint Constraints Profile:** Selecting appropriate joint type classifications, such as defining revolute mechanisms for the active abad, hip, and knee configurations while pinning the foot links as fixed joints, and populating their respective mechanical limit boundaries.

The physical joint constraints were initially extracted and hardcoded based on the absolute mechanical clearances of Scotty’s aluminum frame. The allowable operational boundaries were mapped as: abad limits set to $\pm 35^{\circ }$, hip limits extending from $-70^{\circ }$ to $+100^{\circ }$, and knee rotation limits bounded strictly between $0^{\circ }$ and $-140^{\circ }$. To prevent model failures within the virtual physics environment, the maximum effort threshold was capped at 50 Nm, and the maximum joint velocity boundary was defined as 3 rad/s across all degrees of freedom.

#### 5.1.2. Xacro Enhancement

To improve modularity, eliminate code redundancy, and enable parameterized modifications, the raw exported URDF was manually re-architected into a scalable XML Macro (Xacro) framework. The main top-level Xacro script acts as a central workspace aggregator, compiling distinct modular sub-files containing isolated descriptions for the base body, external sensors, mechanical transmissions, and standardized leg models.

Key architectural components manually appended to the macro definitions include:

- **Floating Joint and World Anchor:** A floating joint connecting the structural base_link to a virtual fixed world frame to enable unconstrained 6-DoF dynamics during Gazebo execution;
- **Sensor Simulation Hardware Plugins:** An integrated Inertial Measurement Unit (IMU) link node combined with an automated Gazebo IMU sensor plugin to stream real-time simulated linear accelerations and angular velocities;
- **ROS Control Integrations:** Unified ros_control packages [26] to manage joint trajectory controllers, simulate localized hardware loop behaviors, and calculate virtual odometry states;
- **Transmission Elements Mapping:** Explicit transmission tag definitions for all active revolute joints, pairing individual software control interfaces with physical motor actuator variables;
- **Surface Contact Dynamics Parameters:** Specialized Gazebo surface contact friction properties ($k_{p},k_{d},\mu_{1},\mu_{2}$) applied to the foot links to prevent slipping and ensure valid ground reaction forces during stance phases.

Crucially, the raw link mass distributions and 3×3 spatial inertia tensors (I) were manually audited and updated within the macro tags using values calculated relative to each individual link’s joint coordinate frame in SolidWorks, ensuring simulation fidelity.

#### 5.1.3. CHAMP Setup Assistant Configuration

The complete, audited Xacro package was imported into the champ_setup_assistant graphic wizard to generate the configuration infrastructure [14]. This step automatically parsed the underlying kinematic chains to validate structural link transformations. The physical limbs were explicitly mapped into CHAMP’s standard layout naming conventions: Front Left (FL), Front Right (FR), Rear Left (RL), and Rear Right (RR).

To ensure the trajectory planner aligned with Scotty’s knee geometry, the knee orientation parameter was set to “.«”. In this notation, the dot ‘.’ represents the front side of the robot, while the arrows represent the bending direction of the front and rear knees respectively (where ‘<’ indicates bending forward towards the front, and ‘>’ indicates bending backward away from the front). Setting this to “.«” indicates that both front and rear knees bend forward, aligning with Scotty’s physical mechanical design. The maximum vertical leg extension limit (clearance) was defined at 0.50 m.

The assistant automatically compiled the final, production-ready directory configuration package. This package aggregates isolated YAML files defining detailed gait parameters, joint and link constraints, move_base navigation costmaps, and ros_control settings, alongside specialized launch files engineered for RViz visualizations, automated Gazebo world environments, and keyboard-based teleoperation controls.

### 5.2. Simulation Environment

#### 5.2.1. RViz Visualization and Kinematic Verification

Following the configuration profile generation, Scotty’s baseline assembly, structural transformations, and joint frames were systematically verified within the Robot Operating System (ROS) visualization suite (RViz). This visual auditing phase was executed by bringing up the localized robot state publisher and model geometry trees using the main framework configuration launch file:

roslaunch scotty_config bringup.launch rviz:=true

The entire physical structure compiled without coordinate link breaks or unmapped geometric transforms, verifying that the physical parameters, coordinate frameworks, and joint transformations derived from the exporter script were accurately translated into the software domain.

**Figure 15:**
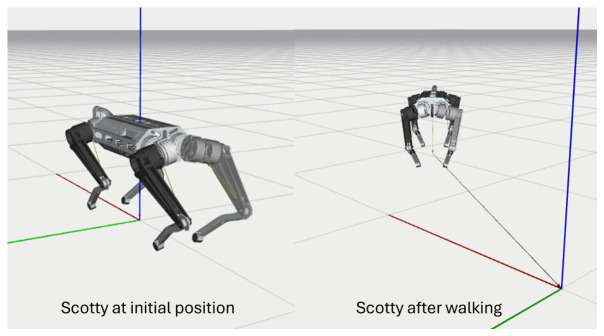
RViz visualization showing Scotty’s initial spawn position (**left**) and position after keyboard teleoperation (**right**).

Active joint mapping and trajectory routing were evaluated via the champ_teleop node, which injects Twist velocity inputs (cmd_vel) directly into the high-level gait planner loop. Under this configuration, Scotty successfully demonstrated fluid link movements and stable foot placement transformations relative to the central base coordinate frame, as shown in Figure 15. This test confirmed the algebraic integrity of the inverse kinematics solver and verified that target velocity values were properly distributed into single-joint position trajectories across all twelve operational channels.

#### 5.2.2. Gazebo Integration and Critical Framework Bottlenecks

**Figure 16:**
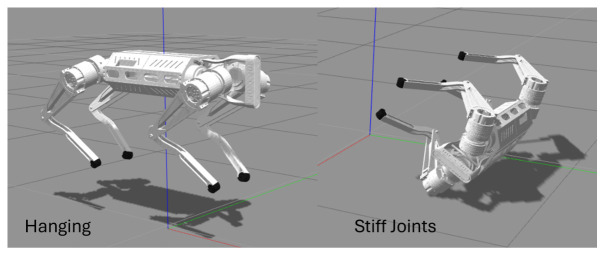
(**Left**) “Stuck in Mid-Air” bug: Robot spawns immobilized and suspended above the ground plane due to a fixed world joint in the URDF erroneously anchoring the chassis to the virtual origin. (**Right**) “Stiff Legs” problem: After fixing the floating joint, legs become rigid and unresponsive because CHAMP’s trajectory node continuously publishes static stand positions, overwriting teleoperation commands. Both issues required URDF re-architecture and state machine intervention to resolve.

Transitioning the system package from a visual coordinate check to a dynamic rigid-body physics simulation environment was achieved by initiating the physical simulation pipeline via the Gazebo launcher sequence (roslaunch scotty_config gazebo.launch). However, executing initial validation tests exposed two critical integration anomalies that directly prevented automated locomotion execution:

1. **The “Stuck in Mid-Air” Bug:** As shown in Figure 16 (left), when spawning into the simulated world, the robot remained completely immobilized and suspended in mid-air above the structural ground plane. Monitoring active transform trees tracked this constraint back to a structural flaw in the raw URDF export, which mistakenly anchored the robot chassis to a fixed, unyielding virtual world origin frame. To restore dynamic physical behaviors, the rigid world joint was removed, and an unconstrained 6-DoF floating joint parameter was mapped between the main base_link frame and the trunk link structure. This re-architecture allowed Scotty to drop naturally and settle onto the dynamic world plane under realistic simulated gravitational forces.
2. **The “Stiff Legs” Problem:** Once grounded on the world plane, the robot’s limbs immediately became completely stiff and unyielding, failing to bend or articulate in response to interactive simulation inputs, as depicted in Figure 16 (right). Analyzing the active network graph using the rqt_graph diagnostic utility revealed that the underlying CHAMP trajectory generation node was continuously publishing static stand position arrays to the joint command topic (/joint_group_position_controller/command), locked at a fixed execution frequency. This continuous stream completely blocked teleoperation inputs by overwriting dynamic movement vectors with static position configurations.

Crucially, while single-gait walking velocity profiles could be generated successfully by the background teleoperation stacks, the base CHAMP framework possessed no internal operational state controller capable of governing transition flow sequences (e.g., navigating from an uninitialized Idle posture, into sitting down (Down), standing up (Stand), or safely engaging walking (Walk) modes). Attempting to pass velocity commands while the robot was unready or in an invalid state induced immediate torque tracking loops, forcing severe structural failures in the physics engine. This critical limitation exposed an important “framework gap,” confirming that safe hardware integration requires a dedicated high-level state machine controller to arbitrate control topic authority and manage stable, sequential operational shifts.

### 5.3. Custom State Controller Design

#### 5.3.1. State Machine Architecture

To manage the operation of the robot safely, a custom high-level state controller was developed as an independent, ROS-based Python 3 node (/scotty_main_controller). This manager acts as a coordinator, deciding which software modules have control over the joints at any given moment.

**Figure 17:**
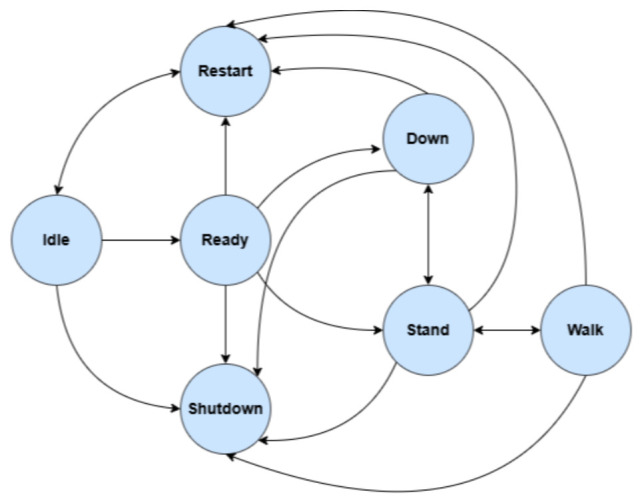
Finite state machine diagram for Scotty’s custom high-level controller showing seven operational states (Idle, Ready, Down, Stand, Walk, Shutdown, Reset) and valid transition paths.

As shown in Figure 17, the robot moves through a clear set of predefined operational states:

- **Idle:** The starting point of the software, where internal parameters are set up and the simulation environment is initialized;
- **Ready:** The system stands by, confirming that all background connections and nodes have loaded successfully;
- **Down:** The robot lowers its body smoothly into a resting position close to the ground;
- **Stand:** The robot pushes up from the ground into an upright, stable standing posture;
- **Walk:** Active locomotion is enabled, allowing the robot to accept movement commands from the CHAMP controller;
- **Shutdown:** A safe exit routine that brings the robot to a low rest before cleanly turning off active nodes;
- **Reset:** Clears the current environment and restarts the simulation cleanly.

To prevent sudden, dangerous joint movements, strict transition guidelines are forced by the software. For example, the robot can only enter the Walk state from the Stand state, and it must decelerate back to Stand before it can exit the walking mode. Transitions to critical safety configurations like Shutdown and Reset, however, are allowed immediately from any state.

#### 5.3.2. Implementation Details

To maintain a modular layout, each state runs as a separate ROS node that is launched on demand using Python’s subprocess.Popen commands. The main controller tracks progress using a simple validation dictionary and locks the system down using an internal "Busy" state while a transition is taking place. This lock prevents overlapping commands from corrupting the joint targets.

Once a state finishes moving the joints, it publishes an explicit confirmation message (such as "Down Done" or "Stand Done") over the status topic (/scotty_controller/state_execution_status). The main manager captures this token, releases the Busy lock, and updates the global system state.

When switching to the Walk state, the manager launches the full CHAMP navigation stack. Leaving the Walk state triggers a systematic termination script that safely shuts down the locomotion nodes (/champ_controller, /state_estimator, etc.) and hands over the leg joints back to direct position control smoothly.

#### 5.3.3. Graphical User Interface

To make operating the state machine intuitive, a web-based dashboard interface was built using HTML, JavaScript, and ROSLIB.js [27], connecting to the robot via a rosbridge WebSocket connection.

**Figure 18:**
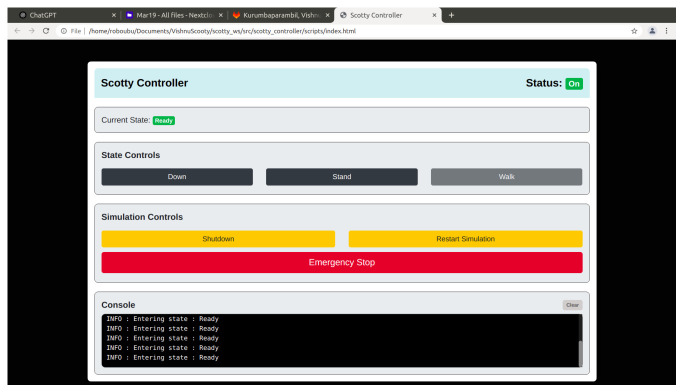
Web GUI for Scotty’s state machine controller built with HTML, JavaScript, and ROSLIB.js via rosbridge WebSocket connection. The interface displays: (top) live connection status and current operational state; (middle) interactive state control buttons (Down, Stand, Walk) with dynamic enable/disable based on valid transitions; (bottom) simulation controls (Shutdown, Reset Simulation) and Emergency Stop override; (lower) color-coded scrolling terminal console with categorized logs (INFO, WARN, ERROR). The GUI locks all controls except Emergency Stop during “Busy” transitions.

The user dashboard, as shown in Figure 18, provides:

- A live connection indicator showing the status (*On/Off*) of the data bridge;
- A clear display showing the active operational state of the robot;
- Interactive state buttons that automatically turn on or grey out based on whether a transition is allowed from the current pose;
- Large, red, single-click override buttons for Shutdown, Reset, and Emergency Stop;
- A color-coded scrolling terminal console displaying live logs categorized into informational messages, warnings, and errors.

Whenever the system enters the Busy configuration during a joint transition, the GUI interface locks out all action inputs except for the Emergency Stop, safeguarding the platform against conflicting inputs.

#### 5.3.4. Safety Mechanisms

**Figure 19:**
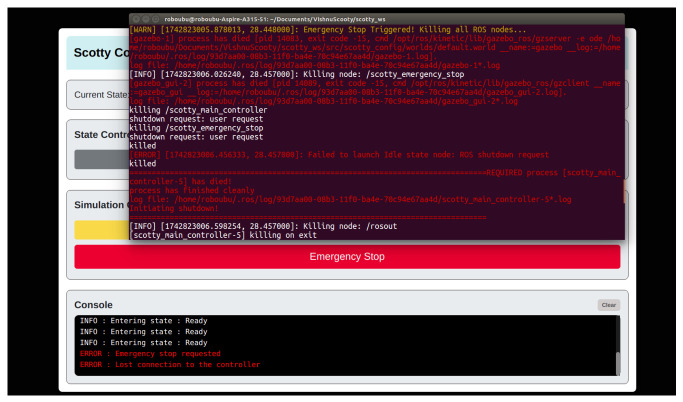
Emergency stop activation sequence showing the web GUI (**left**) and terminal output (**right**) during an E-stop event. The independent emergency node immediately overrides all background threads, kills active ROS nodes, and safely terminates processes to freeze limb positions.

The high-level controller embeds three fundamental safety pillars to ensure hardware and simulation protection:

- **Valid Transition Enforcement:** If a user sends an illegal state request, the controller instantly rejects the message and prints a warning log, leaving the active leg configuration untouched;
- **Emergency Stop Control:** As illustrated in Figure 19, a separate, high-priority emergency node can be called at any point. This script instantly overrides all background threads, cuts command lines, and safely terminates active processes to freeze the limbs immediately;
- **Controller Resource Management:** The controller ensures that joint controllers are properly stopped before new trajectory files are loaded, entirely eliminating topic conflicts.

### 5.4. State Implementation Details and Simulation Results

#### 5.4.1. Idle and Restart States

The operational sequence begins by spawning the robot model directly inside the Gazebo simulation environment. To guarantee a safe start and prevent sudden leg collisions with the environment, default joint configurations are configured prior to unpausing the physics world. This task is handled using a specialized service call:

rosservice call /gazebo/set_model_configuration

To make this process reliable, a dedicated waiting loop script was built to monitor for specific system load markers. The controller continuously checks the ROS topic list and pauses execution until it detects that the core gazebo model topics are completely active and ready.

During this initial startup phase, the Gazebo physics simulation engine is kept strictly in a paused state. This allows the robot to fully load its joint controllers without fighting gravity. After a brief, calculated stabilization delay, a command unpauses the physics simulation, letting Scotty drop slightly and settle naturally onto the ground plane under simulated gravity.

**Figure 20:**
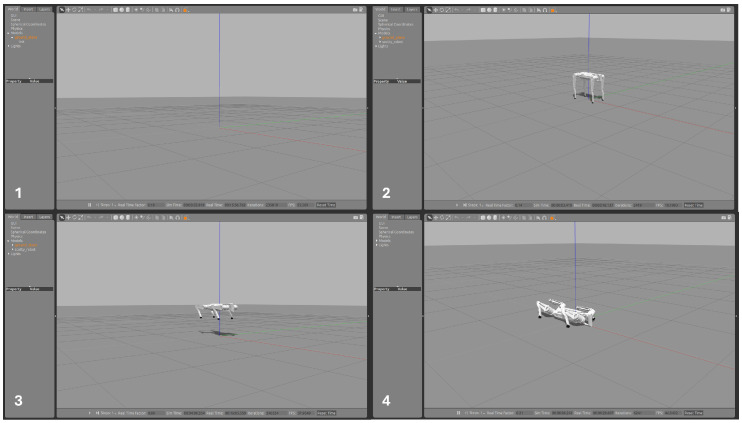
Snapshots of the GUI during a reset.

For the Reset state, a quick-restart pipeline was engineered to clear the workspace without needing to relaunch the main ROS master node. When triggered, the controller invokes the delete_model service to wipe the corrupted robot configuration from the simulator screen, clears out any remaining trajectory messages in the queue buffers, and re-spawns a fresh model instantly via the simulation spawning scripts, as illustrated in Figure 20.

#### 5.4.2. Down and Stand States

When moving between the resting (Down) and upright (Stand) configurations, the main controller relies on standard ROS controller manager services to dynamically load and start the necessary trajectory infrastructure. To achieve fluid, human-like leg adjustments and avoid sudden motor jerks, the joint commands are wrapped inside a time-parameterized trajectory loop.

The system packs target angles into joint trajectory messages and publishes them directly to the controller input topic:

/joint_group_position_controller/command

**Figure 21:**
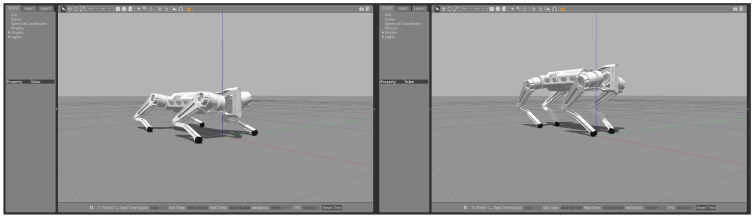
Gazebo screenshots showing smooth posture transitions between Down (**left**) and Stand (**right**) states.

Rather than instantly snapping to a new angle, the controller enforces a smooth three-second execution window. This trajectory profiling divides the angular distance into small increments, enabling Scotty to sit down or stand up in a highly controlled and balanced way. The simulator screenshots during Down and Stand states are shown in Figure 21.

#### 5.4.3. Walk State

Activating the locomotion pipeline triggers the execution of the primary CHAMP launch stack. This script boots up the high-level trajectory calculators, leg inverse kinematics solvers, and the remote teleoperation listener nodes. This configuration allows the operator to steer Scotty around the simulation world in real-time using standard directional velocity commands.

When the user commands the robot to stop walking and return to a static standing posture, a clean handoff routine is essential to prevent joint command conflicts. The controller executes a targeted termination script that systematically shuts down all active CHAMP nodes (/champ_controller, /velocity_smoother, etc.). Once those locomotion loops are dead and their command locks are released, the script cleanly transfers joint resource authority back to the direct group position controllers.

#### 5.4.4. Simulation Results Summary

Through rigorous testing across the complete virtual pipeline, Scotty successfully achieved all primary simulation milestones:

- Accurate visual validation and teleoperation tracking inside RViz;
- Stable gravity-based dropping and unconstrained body balancing inside Gazebo;
- Repeatable, smooth posture transitions between the Down and Stand states without causing controller crashes;
- Real-time steering and gait generation via CHAMP teleoperation controls;
- Instantaneous safety cutoff verification using the independent Emergency Stop node;
- Successful cycling through the complete operational life loop: Idle→Ready→Down→Stand→Walk→Stand→Shutdown.

During walking tests in Gazebo, teleoperation velocity commands were incrementally increased from minimal values. The primary objective of these simulation runs was qualitative functional validation: verifying that inverse kinematics solvers operated correctly, that joint commands from the high-level ROS nodes were mapped in the proper coordinate directions, and that the custom state machine transitions (Idle, Ready, Down, Stand, Walk, Shutdown) operated reliably without system crashes.

Because the simulation was used as a functional verification gatekeeper rather than a high-fidelity dynamic study, active telemetry logging of base attitude variations, height deviations, and foot-slip velocities was not implemented. Minor body bouncing and intermittent foot slip were observed visually during extended runs, which are typical when using standard physics engine defaults. To support reproducibility, the underlying simulation configuration parameters are detailed below:

- **Physics Engine Configuration:** The Gazebo simulation utilized the default Open Dynamics Engine (ODE) solver with a fixed time step of 0.001 s (1kHz physics update rate). The friction coefficient for the rubber foot links in the URDF model was set to a nominal value of $\mu_{1}=\mu_{2}=0.8$ against the ground plane.
- **Joint Controller Configuration:** The simulated joints were actuated using ROS joint position controllers. The proportional–derivative (PD) gains for the abduction (abad) joints were set to $K_{p}=100$ N·m/rad and $K_{d}=0.4$ N·m·s/rad, while the hip and knee joints were configured with $K_{p}=150$ N·m/rad and $K_{d}=1.0$ N·m·s/rad to provide adequate joint stiffness under gravity.

## 6. Hardware Interface Layer

### 6.1. SPI Communication Protocol

The hardware interface layer establishes a reliable, full-duplex Serial Peripheral Interface (SPI) communication pipeline between the high-level x86 UP Board (acting as the master device) and the two low-level STM32 microcontrollers (acting as slave devices). The system peripheral mapping follows strict physical routing configurations: the master addresses the individual sub-controllers using dedicated slave select lines mapped to system nodes /dev/spidev2.0 and /dev/spidev2.1.

A key architectural decision in designing this protocol was the deliberate reuse of the low-level communication infrastructure developed during our initial integration trials with the MIT framework. Because the interrupt-driven SPI firmware, pin remappings, and MISO tri-state safety parameters had already been fully tested, verified, and stabilized on the STM32 microcontrollers, replicating the MIT framework’s data packing scheme offered a highly efficient path forward. Instead of re-engineering the low-level firmware from scratch to accommodate CHAMP’s native formats, the high-level ROS hardware interface node was designed to pack and unpack data structures that perfectly mirror the established MIT communication layout. This strategy enabled a modular reuse of our low-level controller layer, significantly reducing development time while guaranteeing high communication stability.

Consequently, the protocol uses rigid, fixed-size command and state data structures packed identically to the legacy layout:

- **Command Structure (Master-to-Slave):** The UP Board packages high-level trajectory data into a compact array containing the targeted control parameter arrays for the abduction/adduction (abad), hip, and knee joints. For each individual joint, the structure packs the desired position float, target velocity, desired feed-forward torque, proportional gain ($K_{p}$), and derivative gain ($K_{d}$).
- **State Structure (Slave-to-Master):** Concurrently during the same SPI bus clock cycle, the STM32 controllers shift back a state telemetry package containing the physical feedback metrics of each joint. This feedback includes the actual measured joint position, current angular velocity, active motor torque output, and low-level diagnostic status flags.

By using fixed-width data packets that match the pre-tested configuration, the architecture eliminates the communication overhead caused by dynamic string parsing, supporting consistent, error-free packet transmission within the bounds of the reported stress testing.

### 6.2. Standalone SPI Verification

Before binding the communication pipeline to active ROS networks, a standalone C++ verification environment was constructed to evaluate raw hardware connectivity and device enumeration. This evaluation phase isolated the physical SPI buses from high-level software loops to confirm that the hardware buses could sustain clean data transfers without dropping packets.

**Figure 22:**
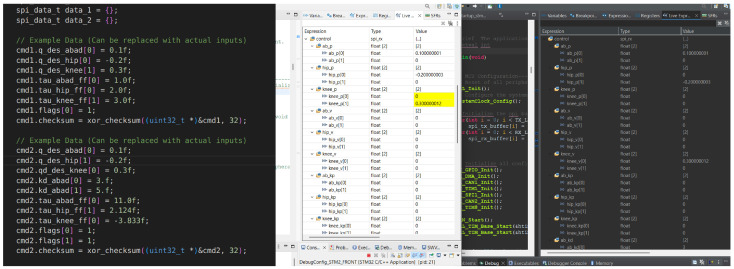
Terminal diagnostics from the UP Board confirming proper SPI device enumeration and master bus register initialization. The test validates device paths /dev/spidev2.0 and /dev/spidev2.1, with debug expressions showing correct data structure packing for command and state variables.

As shown in Figure 22, terminal commands were executed directly on the UP Board to print the data, confirming the correct configuration of the device path and the stable initialisation of the master bus registers.

**Figure 23:**
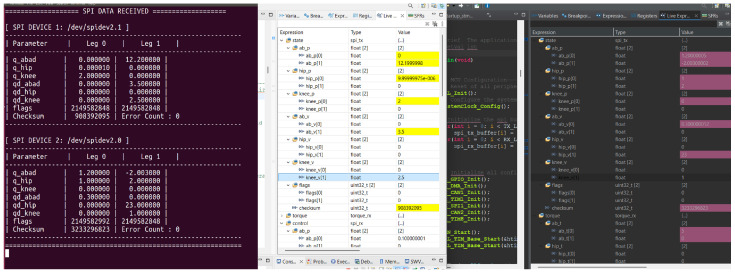
SPI communication validation showing that the master receives test data (**left**) from the STM32 slaves (**right**). Error count remains zero across both SPI devices, validating that there are no checksum errors.

The communication validation test was conducted by streaming dummy byte arrays down the master lines. The low-level microcontrollers were programmed to trap the incoming buffers and immediately echo the identical payload packet back across the MISO channel. As logged in the console outputs displayed in Figure 23, the loopback test achieved a perfect transmission record with zero bitwise drops, validating the integrity of the hardware lines, terminal wiring paths, and slave-select clock gating.

### 6.3. ROS Integration

#### 6.3.1. Simple Subscriber Node

To verify that data could flow correctly from the ROS environment down to the physical SPI lines, a lightweight validation node named the Simple Subscriber Node was developed. This node serves as an isolated testing tool to verify message parsing without running the heavy background calculations of the full locomotion engine. The node listens directly to the primary joint command topic:

/joint_group_position_controller/command

Whenever a standard ROS trajectory message is published, the subscriber catches it, extracts the joint position angles, and maps them directly into our structured SPI command arrays. This isolated test confirmed that the high-level processor could listen to standard ROS commands and repackage them into raw data bytes in real-time without introducing processing lag or buffer overflows.

#### 6.3.2. Hardware Interface Node

Once the basic listening paths were verified, the full communication pipeline was deployed within a centralized Hardware Interface Node. This node serves as the complete, full-duplex data router handling the real-time interaction loop between the CHAMP controller stack and the low-level embedded hardware.

The node continuously executes a five-step communication loop at a steady frequency:

1. **Subscribe:** It listens to the active joint position and velocity commands streaming from the high-level CHAMP walking planner;
2. **Convert:** It maps these dynamic movement vectors into our custom fixed-width SPI command structure, matching the pre-tested MIT data layout;
3. **Transmit:** It drives the hardware lines to send the packed command structures down to the two STM32 leg microcontrollers over the full-duplex SPI bus lines;
4. **Receive:** Concurrently during the same clock cycle, it extracts the incoming state telemetry buffers sent back by the STM32 controllers;
5. **Publish:** It unpacks the raw actuator state parameters (actual positions, velocities, and torques) and translates them back into a standard ROS message, publishing them to the global network over the /joint_states topic.

**Figure 24:**
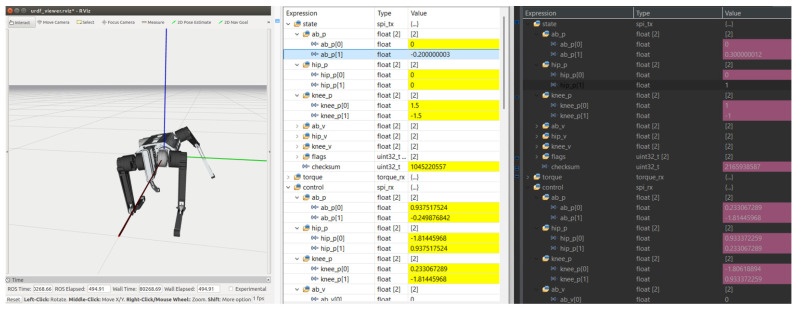
Integrated pipeline validation showing: (**left**) RViz with Scotty model; (**right**) commands received in STM boards from high-level CHAMP controller and also the current state of the motor joint values. The hardware interface node successfully: (1) subscribes to CHAMP joint commands; (2) converts ROS trajectory messages to SPI command structures; (3) transmits to STM32 slaves; (4) receives state telemetry; (5) publishes /joint_states back to ROS. Highlighted values show commanded positions (yellow) matching feedback positions (pink), confirming zero-drop full-duplex operation at steady frequency.

Successful full-duplex operation of the integrated ROS-to-SPI node framework was validated. As seen in Figure 24, the complete pipeline connecting the high-level ROS framework and the low-level embedded hardware is successful.

### 6.4. Joint Control Test GUI

To allow for safe, manual troubleshooting and isolated motor tuning on the physical robot, a dedicated hardware testing GUI was developed. Running the full high-level CHAMP locomotion planner during early hardware testing can be dangerous, as any uncalibrated gait parameters could command sudden, high-torque movements that risk damaging the physical links. This standalone testing GUI completely bypasses the main walking planner, allowing the operator to interact directly with the hardware interface node in a safe and controlled manner.

The diagnostic software environment provides the following core features:

- **Individual Joint Position Publishing:** Operators can select a specific limb and use interactive sliders or input boxes to send precise position commands to a single joint at a time;
- **Real-Time Telemetry Tracking:** The interface listens directly to the feedback streams coming up from the STM32 microcontrollers, displaying real-time plots of actual joint positions, velocities, and motor torque outputs;
- **Isolated Parameter Tuning:** Users can adjust Proportional–Derivative ($K_{p}$, $K_{d}$) control gains on the fly, allowing individual limb behaviors to be fine-tuned before deploying the full-body walking stack.

**Figure 25:**
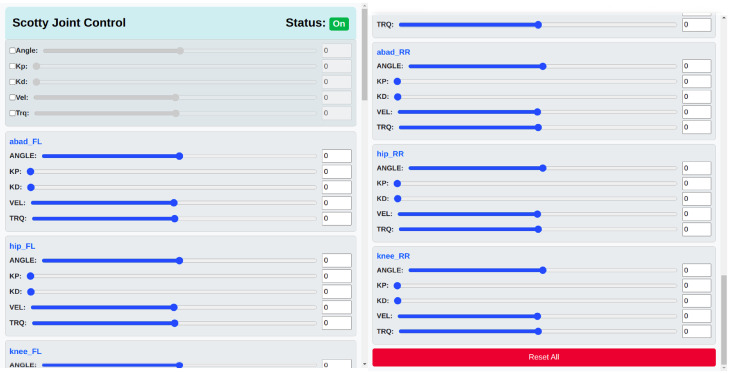
Standalone hardware testing GUI for safe individual joint calibration without running the full CHAMP locomotion stack. The interface provides interactive sliders and input boxes for commanding position, Kp, Kd, velocity, and torque to individual joints (abduction, hip, knee) per leg.

The calibrator GUI is shown in Figure 25.

Once basic mapping was verified, the custom dashboard was connected directly to the physical prototype suspended in the test rig. The calibration tests were highly successful: the physical leg joints responded smoothly to manual position sliders, and the real-time feedback loops perfectly recorded and displayed the physical movement vectors. This successfully validated the integrity of the full-duplex SPI communication link, the hardware CAN routing paths, and the software translation logic under manual supervision.

## 7. Preliminary Hardware Integration and Validation

### 7.1. Extension to Hardware Mode

**Figure 26:**
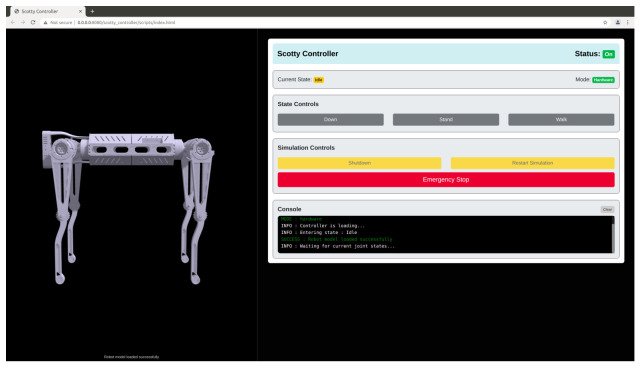
Extended web dashboard operating in physical hardware mode (indicated by green “Hardware” status badge). The interface shows the 3D robot model, current state (Idle), and live console logs tracking the initialization sequence: controller loading, SPI communication establishment, and current joint state acquisition. The hardware mode disables simulation-specific controls (Reset Simulation) while maintaining state controls (Down, Stand, Walk) and Emergency Stop functionality for safe physical operation.

Following the successful validation of the modular control architecture, the custom high-level state controller was systematically extended from the virtual simulation domain [28] to support physical hardware operations on the prototype platform (Figure 26). Transitioning the control loops to the physical quadruped required three primary system modifications:

- **SPI Communication Initialization:** Configuring the userspace spidev interface files via IOCTL system calls to open full-duplex, bidirectional communication channels over the master device paths /dev/spidev2.0 and /dev/spidev2.1.
- **Clock and Date Synchronization:** Implementing an explicit temporal alignment protocol between the external ground station computer and the onboard x86 UP Board single-board computer to eliminate persistent timestamp warnings within the ROS master node network.
- **Safe Startup Sequence Execution:** Structuring a defensive initialization state machine to actively detect, intercept, and manage invalid initial actuator feedback states during the initial power-on sequence.

### 7.2. Validation Results

The empirical milestone progression achieved during the physical deployment phase is summarized in Table 3, providing explicit verification metrics for individual subsystems.

To rigorously evaluate the reliability of the communication bridge and joint actuation, quantitative stress tests and calibration trials were conducted:

- **SPI/CAN Bridge Reliability:** Bidirectional full-duplex communication was operated at a loop frequency of 100 Hz. To verify stability after implementing the firmware-level MISO select tri-state fix (resolving bitwise OR bus collisions), stress tests were conducted. In the two longest uninterrupted runs, the interface successfully processed 197,020 packets (≈32.8 min of continuous operation) and 116,408 packets (≈19.4 min of continuous operation) without a single checksum mismatch or packet drop (0.00% packet error rate). The CAN bus was operated at 1 Mbit/s, enforcing a deterministic 300 μs inter-message transmission delay between successive joint commands to prevent network congestion.
- **Joint Tracking Calibration Errors:** Localized joint-level movement tests were performed using a GUI calibration slider to map command inputs to actual actuator motion. Tracking errors ($\Delta q=|q_{\mathrm{des}}-q_{\mathrm{act}}|$) were computed offline from logged feedback telemetry, as summarized in Table 4. The hip and knee joints exhibited high tracking precision (mean errors ≤0.0976 rad), whereas the abduction/adduction (abad) joint showed a higher mean error range (0.1628 to 0.2517 rad) and standard deviation (0.2835 rad) due to structural backlash in the physical leg mounting brackets.

### 7.3. Issues Encountered

**Figure 27:**
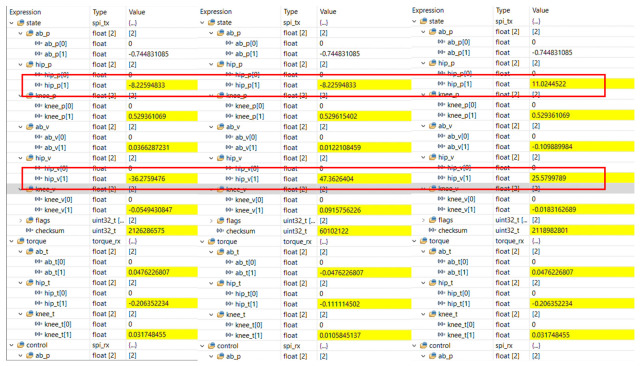
Debug expression showing anomalous telemetry from the hip motor controller. These intermittent high-frequency spikes indicate communication issues, encoder noise, or hardware instability within the motor actuator itself, representing a key limitation of the current physical validation phase. This issue requires startup validity checks and data filtering to prevent erratic command execution.

During hardware integration testing, the following system bottlenecks and anomalous communication behaviors were identified and evaluated:

- **Old Values Warning and Time Synchronization:** The central ROS network generated continuous system warnings regarding outdated values received over the global /joint_states topic. This temporal mismatch was resolved via a practical workaround by forcing an interactive date synchronization command from the ground control PC over the local network to align clocks with the onboard x86 UP Board:
        sudo date --set "$(ssh scotty@192.168.2.40 ’date -u’)"
  where 192.168.2.40 represents the static IP address of the UP Board. Testing verified that rigorous time-matching is mandatory to ensure correct sequential message handling and to prevent joint state TF transforms from being dropped by the high-level ROS navigation stack. While this manual alignment served as a practical workaround for the current prototype setup, a more robust and automated synchronization method (such as utilizing Chrony or NTP daemon protocols over a local server) would be preferable in future production-grade hardware deployments.
- **Hip Motor Abnormal Values:** Intermittent high-frequency data spikes and unusual numerical readings were observed coming from the hip motor controller under active test cycles, which are currently being analyzed under localized diagnostics (Figure 27). These abnormal readings, which could stem from communication interference, encoder noise, or instability/defects within the motor actuator hardware itself, represent a key limitation of the current hardware validation phase. They prevent reliable feedback tracking and pose risks to closed-loop stability, restricting physical testing to static or localized joint movements. To mitigate this in the current prototype, validity checks were performed at startup to ensure that the motor states are within the expected range, and future work will require dedicated hardware-level filtering, actuator debugging, and diagnostic troubleshooting.

### 7.4. Safe Start Procedure

**Figure 28:**
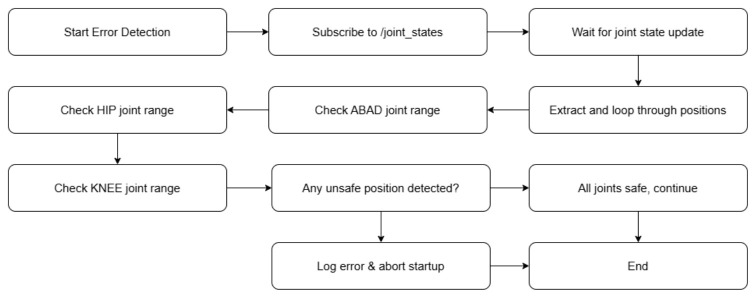
Algorithmic validation sequence for safe motor startup. The procedure: (1) starts error detection; (2) subscribes to /joint_states; (3) waits for joint state update; (4) extracts and loops through all joint positions; (5) validates each joint (abduction, hip, knee) against realistic physical limits; (6) if any unsafe position detected, logs error and aborts startup; (7) if all joints safe, confirms startup and proceeds to normal operation. This defensive loop protects against invalid initial actuator feedback that could cause high-torque tracking errors during power-on.

Because the platform’s brushless DC motor controllers occasionally transmit invalid or unstable data packages when first powered on, a formalized Safe Start Procedure was designed and embedded directly within the initialization firmware (Figure 28).

The sequence processes through the following algorithmic steps:

1. Issue the specific CAN code frame to command the actuators to enter motor control mode;
2. Intercept and read the incoming raw motor feedback states across the parallel CAN bus networks;
3. Validate the received data packets by verifying that the joint position, velocity, and torque parameters fall strictly within expected, realistic physical limits;
4. If any data point is flagged as invalid or unparsable, reject the packet immediately and retry the loop up to a configured maximum number of attempts;
5. If the incoming telemetry completely satisfies the validation parameters, confirm the startup status and proceed to normal system operation;
6. If the maximum number of retries is exceeded without receiving valid data, halt the process immediately, isolate the actuators, and enter a safe error state.

This validation sequence successfully intercepts and corrects most power-on communication errors, outputting clear verification confirmations when valid states are restored. However, because subtle, intermittent invalid states can still occasionally slip through during runtime, resolving these persistent synchronization issues and tuning the motor startup timing represent key technical objectives for future work.

## 8. Discussion: Lessons Learned and Recommendations

### 8.1. Framework Selection Paradigms

When developing custom quadrupedal platforms under restricted timelines, code modularity and structural documentation prove far more valuable than theoretical high-dynamic agility. The primary selection baseline evaluated in this work revealed a clear contrast: while the MIT Mini Cheetah framework offers unmatched potential for dynamic torque control, its tightly coupled software blocks and lack of baseline documentation required months of low-level embedded reverse-engineering. Conversely, the CHAMP framework’s clean organization, automated setup assistant, and native ROS compatibility allowed a complete, visualizer-validated locomotion model to be deployed within weeks. For researchers and independent custom platform developers, the choice of a high-level framework impacts project timeline viability more than the complexity of its underlying control algorithm.

### 8.2. Simulation-First Development Boundaries

Validating Scotty within a physics simulation engine (Gazebo) was an essential prerequisite to ensure code safety, allowing the identification of structural URDF flaws and enabling the risk-free development of our top-level state machine controller. However, simulation validation alone is structurally insufficient to ensure seamless real-world deployment.

Operating on physical hardware unmasked several operational constraints that never appeared in the idealized simulation loop:

- **Network Packet Timing Gaps:** Simulation handles data transfers perfectly, but physical hardware introduces communication lag and processing queue delays.
- **Clock Desynchronization Errors:** Small timing differences between the operator’s ground control station and the onboard UP Board single-board computer generated immediate timestamp errors in the ROS network, requiring active date-matching loops.
- **Physical Locomotion Constraints:** Shifting from simulation to hardware requires a highly disciplined, incremental validation sequence: moving sequentially from single-joint validation, to full-limb benchmarking, suspended rig testing, and finally ground-contact closed-loop locomotion tuning.

### 8.3. The Engineering Overhead of Motor Startup Loops

A core engineering realization of this study is that designing a safe, repeatable motor startup routine requires just as much programming and development effort as building the locomotion control engine itself. Standard control frameworks operate under the idealized assumption that brushless actuators initialize to a known, stable feedback configuration at power-on.

Physical deployment disproved this assumption, as the AK10-9 actuator drives frequently returned invalid or corrupted data packages upon initial power delivery. Neglecting to implement defensive software checking loops to catch these starting faults introduces severe high-torque tracking loops that can break 3D-printed brackets and cause unwanted movements. Robust telemetry validation, error-catching algorithms, and automated retry parameters are mandatory requirements to protect custom robotic hardware against power-on command spikes.

### 8.4. Open Science and Community Contributions

The field of experimental robotics suffers from a reporting bias, where successful locomotion walk profiles are published while the immense debugging efforts, integration roadblocks, and configuration failures remain unmentioned. If comprehensive troubleshooting notes regarding the MIT framework’s embedded layers or spidev address configurations had been accessible beforehand, months of duplicate debugging effort could have been avoided.

To support reproducibility and accelerate future development cycles for custom quadruped platforms, the complete codebase and integration data are hosted openly under the permissive MIT License at https://github.com/Vichu95/Scotty (accessed on 22 July 2026) (branch version main). The repository contains the modified MIT Mini Cheetah control package, the CHAMP-based ROS nodes, and the custom low-level microcode. Specifically, it includes the SolidWorks-exported URDF/Xacro kinematic files, high-level state machine nodes, the C++ SPI-to-CAN hardware interface, STM32 microcode for SPI-mimicking communication, and the raw telemetry and calibration logs from our experimental testing. To keep this manuscript focused on architectural and integration lessons, directory structures and command-line execution steps are omitted here; instead, a comprehensive README.md is provided in the repository to guide developers through catkin workspace compilation, Gazebo simulation startup, and SPI communication verification loops.

### 8.5. Recommendations for Future Builders

Based on the integration logs and framework characteristics evaluated throughout this project, future developers can select an engineering path tailored to their specific resources and research objectives. For projects focused on custom hardware fabrication, automated navigation stacks, or those operating under restricted timelines, deploying the CHAMP framework represents the most practical approach, as its extensive documentation and native ROS compatibility drastically reduce deployment risks. Conversely, the MIT Mini Cheetah framework is best reserved for scenarios where developers plan to replicate the proprietary MIT hardware ecosystem exactly, or for research groups explicitly focused on low-level torque-control loops and embedded reverse-engineering. Ultimately, builders must balance their theoretical performance goals against their available development timeline and engineering resources before committing to a control architecture.

## 9. Conclusions

This paper documents the developmental engineering cycle of bringing the custom quadruped robot, Scotty, from initial physical assembly to simulation-validated locomotion and preliminary hardware-interfacing verification. We contribute:

1. A transparent, systematic assessment of the low-level architectural assumptions and integration barriers encountered when attempting to adapt the tightly coupled MIT Mini Cheetah framework to non-standard hardware ecosystems;
2. A complete ROS-based control framework using the CHAMP locomotion engine enhanced with a custom, top-level finite state machine controller to handle operational state transitions missing in the base package;
3. A functional hardware interface layer design that establishes a reliable, full-duplex SPI communication pipeline between the high-level computer and the low-level STM32 microcontroller motor drivers;
4. A practical hardware integration methodology that explicitly maps out hardware-mode constraints, date-synchronization requirements, and power-on validation protocols.

The development framework presented explicitly distinguishes between simulation-validated control loops and preliminary hardware interface deployments. While stable closed-loop hardware locomotion stands as a primary target for future research, the initialization routines engineered here successfully solve motor startup telemetry errors and validate localized leg commands through a custom debugging interface. Documenting and reporting these low-level synchronization limitations precisely ensures that future builders can build directly upon verified software and hardware layers rather than repeating extensive debugging cycles.

All engineering designs, software configurations, localized troubleshooting logs, and the foundational text of this research are hosted openly within our public access repository (https://github.com/Vichu95/Scotty) (accessed on 22 July 2026). We report these limitations precisely so future efforts can build on verified components rather than repeat debugging. Future investigative efforts will focus on refining motor startup synchronization to achieve full body-weight support, followed by extensive gait parameter tuning to establish stable real-world walking partners.

## Figures and Tables

**Table 1:** Comparison of MIT Mini Cheetah and CHAMP frameworks (qualitative entries represent experience-based findings from this development project).

Feature	MIT Mini Cheetah	CHAMP
Control type	Torque-based PD	Position-based trajectory
Documentation	Minimal	Extensive
ROS integration	None	Native
Real-time kernel	Required	Not required
Hardware abstraction	Custom SPIne	ROS Control standard
Simulation	Custom Qt-based	Gazebo/RViz
Community support	Limited	Active
Integration effort for custom hardware	Very high	Moderate

**Table 2:** Scotty quadruped robot technical specifications.

Parameter	Specification/Value
Total physical mass (stripped config)	19.31 kg
Chassis dimensions (L × W × H)	400×250×150 mm
Total foot span (L × W)	740×407.6 mm
Degrees of Freedom (DoF)	12 (3 active joints per leg)
Abad link length/offset	0.070 m
Thigh (hip) link length	0.3175 m
Shin (knee) link length	0.3375 m (including foot radius)
Actuators	12× CubeMars AK10-9 V2.0 KV60 brushless DC motors
Actuator reduction ratio	9:1 (planetary gear)
Knee transmission ratio	1.5:1 (chain and sprocket, total knee reduction 13.5:1)
Peak torque per motor	48 Nm
Continuous torque limit	18 Nm
Motor torque constant ($K_{t}$)	0.198 Nm/A
Phase-to-phase resistance	195 mΩ
Back-drive torque	0.8 Nm
Power supply voltage	24 V nominal (operating range 24–48 V)
Control loops frequency	SPI: 100 Hz; CAN: 100 Hz; ROS: 100 Hz
Joint angular limits	Abad: $\pm 35^{\circ }$ (−0.611 to +0.611 rad); Hip Pitch: $-70^{\circ }$ to $+100^{\circ }$ (−1.222 to +1.745 rad); Knee Pitch: $0^{\circ }$ to $-140^{\circ }$ (0 to −2.443 rad)
Control modes	Joint Position Control (CHAMP)/Joint Torque Control (MIT attempt)

**Table 3:** Hardware integration status summary.

Aspect	Status	Evidence
SPI communication	Validated	100 Hz loop, 0% checksum error over stress tests of 197,020 packets (≈32.8 mins) and 116,408 packets (≈19.4 mins)
Motor command mapping	Validated	Desired joint positions match physical leg movements; mean tracking error ranges ≤0.2517 rad
Motor state feedback	Validated	100 Hz position, velocity, and torque telemetry successfully received over CAN (1 Mbit/s speed, 300 μs delay)
Safe startup sequence	Implemented	Startup data validity filters and automated retry sequence (max 5 retries) verified
CHAMP walking on hardware	Not yet achieved	Motor synchronization issues at startup under full system weight require further parameter tuning
Full closed-loop locomotion	Future work	Physical walk bounded by project time constraints and dynamic parameter calibration limits

**Table 4:** Joint-level position tracking calibration errors ($\Delta q\;=\;|q_{\mathrm{des}}-q_{\mathrm{act}}|$) measured on physical hardware.

Joint	Mean Tracking Error Range [rad]	Standard Deviation [rad]
Abduction/Adduction (Abad)	0.1628–0.2517	0.2835
Hip Pitch	0.0026–0.0281	0.0735
Knee Pitch	0.0105–0.0976	0.1754

## Data Availability

All data, code, and designs are available at https://github.com/Vichu95/Scotty (accessed on 22 July 2026) [29].

## Abbreviations

The following abbreviations are used in this manuscript:

ROS	Robot Operating System
URDF	Unified Robot Description Format
Xacro	XML Macros
SPI	Serial Peripheral Interface
CAN	Controller Area Network
IMU	Inertial Measurement Unit
LCM	Lightweight Communications and Marshalling
GUI	Graphical User Interface
CHAMP	CHAMP Quadruped Robot Framework
MIT	Massachusetts Institute of Technology
STM	STMicroelectronics Microcontroller
NSS	Slave Select
MISO	Master In Slave Out
MOSI	Master Out Slave In
SCK	Serial Clock
PID	Proportional–Integral–Derivative
PD	Proportional–Derivative
FR	Front Right
FL	Front Left
RR	Rear Right
RL	Rear Left
BR	Back Right
BL	Back Left
HTML	Hyper Text Markup Language
API	Application Programming Interface
CSV	Comma Separated Values
CAD	Computer Aided Design
RT	Real-Time
PREEMPT	Pre-emptive scheduling
UART	Universal Asynchronous Receiver–Transmitter
